# Short-Term Effectiveness and Safety of Biologics and Small Molecule Drugs for Moderate to Severe Atopic Dermatitis: A Systematic Review and Network Meta-Analysis

**DOI:** 10.3390/life11090927

**Published:** 2021-09-06

**Authors:** José-Juan Pereyra-Rodriguez, Sara Alcantara-Luna, Javier Domínguez-Cruz, Manuel Galán-Gutiérrez, Ricardo Ruiz-Villaverde, Samuel Vilar-Palomo, Jose-Carlos Armario-Hita

**Affiliations:** 1 Dermatology Department, Hospital Universitario Virgen del Rocío, 41013 Sevilla, Spain; javierj.dominguez.sspa@juntadeandalucia.es; 2 Medicine Department, University of Seville, 41004 Sevilla, Spain; 3 Dermatology Department, Hospital Juan Ramón Jiménez, 21005 Huelva, Spain; sara.alcantara.sspa@juntadeandalucia.es; 4 Dermatology Department, Hospital Universitario Reina Sofia, 14004 Córdoba, Spain; manuel.galan.sspa@juntadeandalucia.es; 5 Dermatology Department, Hospital Universitario San Cecilio, 18016 Granada, Spain; ricardo.ruiz.villaverde.sspa@juntadeandalucia.es; 6 Department, Faculty of Nursing, Physiotherapy and Podiatry, Sevilla University, 41004 Sevilla, Spain; svilar@us.es; 7 Dermatology Department, Hospital Universitario Puerto Real, University of Cádiz, 11003 Cádiz, Spain; josecarlos.armario@uca.es

**Keywords:** atopic dermatitis, meta-analysis, biological agents

## Abstract

Background: Some Network Meta-analysis (NMA) has been published regarding atopic dermatitis (AD). These studies have considered drugs under investigation both in monotheraphy or in combination with topical corticosteroids, as well as systemic immunosuppressant therapies. The objective of this study is to evaluate the efficacy and safety of biological agents and small molecules in AD. Methods: A systematic review and NMA of biologics agents and small molecules in AD was performed. A literature search was performed using MEDLINE, EMBASE, and the Cochrane Central Register of Controlled Trials for clinical trials and systematic reviews between January 2000 and 19 December 2020. Only randomized clinical trials (RCTs) were included. It was limited to English language and adult human subjects. Two networks were evaluated: monotherapy and combination with TCS. The two primary outcomes were Eczema Area and Severity Index (EASI) 75 and EASI 90 change from baseline to week 12–16, depending on source study cut-off. The Cochrane’s Risk of Bias tool 2011 update was used to analyze the risk of bias, focused on the primary objectives. Results: 30 RCTs (included in 26 publications) were included in the systematic review. Finally, 23 RCTs were included in the quantitative analysis (14 RCTs including 3582 patients in monotherapy; and 9 RCTs including 3686 patients with TCS). In monotherapy, a higher percentage of patients achieving EASI-75 was obtained with Upadacitinib 30 mg [OR: 18.90 (13.94; 25.62)] followed by Abrocitinib 200 mg [OR = 11.26 (7.02; 18.05)] and Upadacitinib 15 mg [OR: 10.89 (8.13; 14.59)]. These results were also observed in studies where the use of topical corticosteroid (TCS) was allowed (OR Upadacitinib 30 mg = 9.43; OR Abrocitinib 200 mg = 6.12; OR Upadacitinib 15 mg = 5.20). Regarding IGA, the percentage of patients achieving IGA0/1 was higher with both doses of Upadacitinib 30 mg [OR: 19.13 (13.14; 27.85)] and 15 mg [OR = 10.95 (7.52; 15.94). In studies where the use of TCS were allowed, however, the dose of Abrocitinib 200 mg [OR = 6.10 (3.94; 9.44)] showed higher efficacy than Upadacitinib 15 mg [OR = 5.47 (3.57; 8.41)]. Regarding safety, the drugs with the highest probability of presenting adverse effects were the Janus kinases (JAK) inhibitors, Upadacitinib and Abrocitinib in monotherapy and Baricitinib in combination with TCS. Discussion: Some risks of bias have been found, which must be taken into account when interpreting the results. The funnel plot shows a possible publication bias that may underestimate the efficacy of drugs. Upadacitinib and Abrocitinib are the drugs with the highest efficacy, both in monotherapy and in association with TCS. However, they were also those associated with the highest risk of adverse effects, showing monoclonal antibodies better safety profile. Limitations: We have included molecules still in the development phase as well studies completed and presented at conferences and with data available in Trialsgov^®^ but not published yet. Several molecules’ development had included a small number of patients from 12 to 17 years of age, without being able to differentiate the results from the adult population. Other: Founding: None. PROSPERO database registration number CRD42021225793.

## 1. Introduction

Atopic dermatitis (AD) is a chronic inflammatory dermatosis, genetically based, associated with multiple triggers and complex pathophysiological mechanisms. Its clinical expression is highly heterogeneous, both in the age of presentation and in the signs and symptoms. The defining feature of the disease is the presence of eczema, accompanied by intense itching and dry skin, which leads to an alteration in the barrier function and dysfunction of the immune response towards a T2 response [1].

AD is a pediatric-onset disease in most cases, affecting 10–25% of children and 2–8% of adults in occidental countries. However, it is estimated that approximately 25% of adults with AD may have an onset of the disease in adulthood [2]. Almost 20% of patients have a moderate-to-severe disease [3]. The incidence is higher in women, although in childhood it predominates in men [4].

Topical treatment with topical corticosteroids (TCS) is the milestone of AD treatment. In moderate to severe patients, systemic immunosuppressants, as cyclosporine and azathioprine, are widely used. Recently, Dupilumab, a humanized monoclonal antibody directed against the α subunit of the IL-4 receptor, shared with IL-13, has been incorporated into our therapeutic arsenal. It works by blocking the IL-4/IL-13 receptor/ligand system. It is the first biological therapy for the treatment of this disease, with encouraging results [5]. Tralokinumab and Lebrikizumab are both monoclonal antibodies directed exclusively against IL-13, unlike Dupilumab. Both work by competitively blocking IL-13 from binding to its receptor subunits on B cells and monocytes, thus preventing signal transduction. Nemolizumab is a humanized monoclonal antibody against IL-31, a TH2 cytokine also known as an “itch cytokine”.

Apremilast is an oral phosphodiesterase-4 (PDE-4) inhibitor. Elevated (PDE-4) activity in AD skin compared to normal skin has been well documented. Tezepelumab is a fully human immunoglobulin G2k monoclonal antibody that binds thymic stromal lymphopoietin, an epithelial cell-derived cytokine inducing production of type 2 cytokines, IL-4, IL-5, IL-13, and tumor necrosis factor alpha (TNF-a), by dendritic cells.

Janus kinases (JAK) are enzymes that phosphorylate the domain intracellular of various cytokine receptors. JAK antagonists (antiJAKs) are oral molecules that block signaling to inhibit cytoplasmic receptors. Several JAK inhibitors have shown efficacy in atopic dermatitis. Baricitinib is a selective JAK1/2 inhibitor. Both Abrocitinib and Upadacitinib are selective Janus kinase 1 (JAK1) inhibitors, which reduces interleukin-4 and interleukin-13 signaling.

Since patients with moderate to severe AD require lifelong treatment, reliable evidence regarding the comparative benefits and harms of the interventions is needed to make clinical decisions regarding their use. Meta-analysis are performed to assess the strength of the recommendations and the quality of the available evidence for disease and multiple treatment alternatives, improving the precision of effect estimates and answering questions not raised by individual studies [6]. In network meta-analyses (NMAs), multiple treatments can be compared, connecting evidence from clinical trials that have investigated two or more treatments. The resulting trial network may allow estimation of the relative effects of all pairs of treatments, taking into account direct and indirect evidence [7]. For this reason, among physicians, guideline writers, and health technology agencies, this type of evaluation is gaining strength as an evidence for new interventions.

So far, some NMAs have been published in AD, including randomized trials involving drugs under investigation both in monotherapy or in combination with TCS in the meta-analysis. Meta-analyzes have also considered classic systemic drugs and new therapeutic agents that are not going to be used commercially. The last developed molecules, such as Abrocitinib or Upadacitinib, have not been able to be included in previous NMA. The arrival of new therapeutic tools to address severe AD patients makes a new NMA necessary in order to support decision-making for specialists treating AD. Therefore, we have decided to focus NMA on two different networks, one including the drugs in monotherapy, and the other in combination with TCS.

## 2. Materials and Methods

### 2.1. Search Strategy and Inclusion Criteria

This study is a systematic review of clinical trials assessing the efficacy and safety of biological or small molecules drugs in moderate-to-severe atopic dermatitis. Treatments evaluated included Dupilumab, Nemolizumab, Lebrikizumab, Tralokinumab, Baricitinib, Abrocitinib, Upadacitinib, Tezepelumab, and Apremilast. The study was conducted following the Cochrane Handbook for Systematic Reviews of Interventions Version 5.1.0. [8]. This publication has followed the Preferred Reporting Items fer Systematic Reviews and Meta-Analyzes (PRISMA) statement. Besides, it has been registered in the PROSPERO database with the number CRD42021225793.

A comprehensive literature search was performed using MEDLINE and EMBASE and the Cochrane Central Register of Controlled Trials for clinical trials and systematic reviews using the following set of keywords: [“Dermatitis, Atopic” [MeSH] OR ‘Atopic Dermatitis’] AND [Dupilumab OR Nemolizumab OR Lebrikizumab OR Tralokinumab OR Baricitinib OR Abrocitinib OR Upadacitinib OR Tezepelumab OR Apremilast] AND [‘human’/de].

A literature search of electronic databases was performed for studies published between January 2000 and 19 December 2020. It was limited to English language and human subjects.

### 2.2. Study Selection

Initial screening was based on titles and abstracts by three independent researchers (SAL and JDC). Abstracts lacking information were retrieved for full-text evaluation. Subsequently, the same investigators independently evaluated full-text articles and determined eligibility. Disagreement was resolved by consensus discussions. If it persisted, a third investigator (JPR) decided. Authorship, journal, or years were not blinded.

Only published articles or accepted original studies to international meetings in English were included. Studies examining adult patients (over 18 years) with moderate to severe atopic dermatitis, specifically those with results at 12–16 weeks, were included. This date represents the end of the follow-up period for the different drugs included (12 weeks for Abrocitinib and 16 weeks for the rest) in the pivotal trials. Because some drugs have carried out RCTs, including both adults with adolescents, studies with adults and adolescents (≥12 years) were also taken into account, indicating the percentage of adolescents.

### 2.3. Outcomes

The primary outcomes will be the number of patients who responded to treatment at 12–16 weeks, defined as (a) EASI 75 (reduction of at least 75% from baseline on the EASI scale); (b) EASI 90 (reduction of at least 90% from baseline on the EASI scale).

The secondary outcomes will be the number of patients who responded to treatment at 12–16 weeks, defined as (a) IGA 0 or 1 (patients with IGA 0 (clear) or 1 (almost clear)); (b) 4-point improvement in itch NRS; (c) number of patients experiencing at least one AE; (d) number of patients experiencing at least one SAE.

### 2.4. Data Extraction

Three investigators conducted data extraction following standardized criteria. The following data were extracted: author, year of publication, drug under research, dosing, study duration, gender (number of males), race (number of Caucasians), age at baseline (average), weight at baseline (average), disease duration (average), baseline Eczema Area and Severity Index (EASI) score (average), baseline Body Surface Area (BSA) score (average), number of patients achieving EASI 50 at weeks 12–16, number of patients achieving EASI 75 at weeks 12–16, number of patients achieving EASI 90 at weeks 12–16, number of patients achieving Investigator Global Assessment (IGA) 0/1 at weeks 12–16, Dermatology Life Quality Index (DLQI) (average) at weeks 12–16, number of patients achieving 4-point improvement in itch Numerical Rating Score (NRS) at weeks 12–16; number of patients with at least one adverse event (AE), number of patients with at least one serious EA (SAE), number of patients with at least one infectious AE, number of patients with at least one upper respiratory tract infection, and number of patients withdrawing the drug under investigation due to AE. In the licensed molecules, only the arms with the commercially approved doses were included. In those molecules not approved but with phase 3 clinical trials, the doses used in phase 3 trials were included. Finally, for those with no completed phase 3 trials but ongoing, doses from these trials published in phase 2 were taken into account.

### 2.5. Risk of Bias Assessment

The Cochrane’s tool for assessing risk of bias in randomized trials 2011 update [8] was used to analyze and make a graphical representation of the risk of bias for each of the included studies. The same three authors carried out this analysis, focusing on the primary objectives (EASI 75/90). This tool analyzes the risk of bias for six specific domains: selection bias, performance bias, detection bias, attrition bias, reporting bias, and other biases. The assessment of these biases determines three established risk levels: high risk (red color), low risk (green color), unclear risk yellow color)). All are represented in two graphs under the labels “Graph of risk of bias” and “Summary of risk of bias” (“between the studies”).

### 2.6. Strategy for Data Synthesis

If studies were sufficiently homogeneous, both frequentist and Bayesian NMA were performed. For binomial variables, odds ratio was calculated (for the total number of randomized patients) and mean was assessed in continuous variables.

The network graph presents the connection status of the studies. In addition to the presentation of the network plot, a qualitative description of the network geometry was made. Statistical heterogeneity was tested using the χ² test (significance level: 0.1) and I² statistic. If high levels of heterogeneity among the trials exist (I² >= 50% or *p* < 0.1), the study design and characteristics in the included studies were analyzed. Due to the fact that the heterogeneity values are not known before carrying out the meta-analysis, we have made a random effects model because it is more conservative. We have tried to explain the source of heterogeneity by subgroup analysis or sensitivity analysis. We omitted studies that were judged to be at high risk of bias and trials with fewer than 50 patients per treatment arm. Net splitting was performed to evaluate the inconsistency. This method splits our network and estimates the contribution of direct and indirect evidence. *p* values < 0.05 implies that there is a significant disagreement (inconsistency) between the direct and indirect estimation.

To carry out the NMA, the netmeta [9] and gemtc R-package [10] were used. The R script used is provided in the Supplementary Material.

## 3. Results

### 3.1. Results of the Search

**Figure 1:**
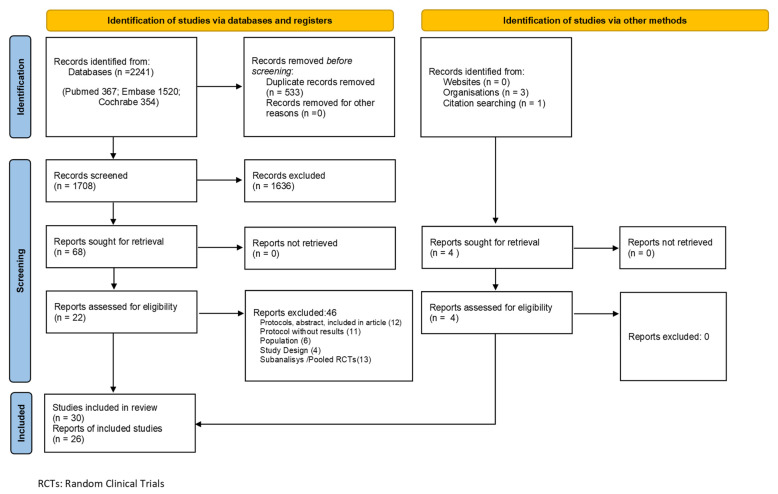
Flow of information through the different phases of the systematic review. RCT: Random Clinical Trial.

In the systematic review process carried out, 2241 records were initially identified but only 72 were assessed for eligibility by meeting the inclusion criteria, and 26 were therefore included (containing 30 RCTs) in the qualitative analysis after the exclusion regarding the reasons exposed in Figure 1, and 19 records (23 RCTs) in quantitative analysis. 7 RCTs did not measure the variables in the defined way and/or did not study the doses evaluated (Table S4). Apremilast only has a phase 2 RCT, whose efficacy data are expressed in numerical form, not as a response rate, so they could not be incorporated in the quantitative analysis. Regarding all the studies analyzed, 14 of them evaluated the drug against placebo and 9 allowed the concomitant use of TCS with the drug versus placebo.

In the clinical trials that only evaluated the study drug in monotherapy versus placebo, 6582 patients were randomized. On the other hand, in those in which the use of TCS was allowed together with the drug, 3686 patients were evaluated. Table 1 shows the included studies, patients by arm, treatment and dose used, as well as the baseline characteristics. Table S1 in the Supplementary Material shows the responses achieved in the different variables analyzed in this study.

The sample size of the clinical trials analyzed ranged between 55 to 603 on those where only the drug was evaluated and a range of 37 to 315 in case the use of TCS was allowed.

**Figure 2:**
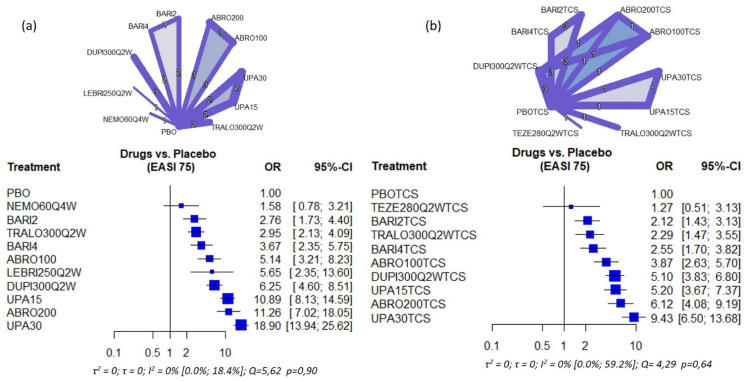
Network plot and Forest plot of the EASI 75 results obtained in the NMA (**a**) Monotherapy (**b**) Combination with topical corticosteroids (TCS). The number in the lines that join each intervention represents the number of studies comparing both drugs; the thickness of the line is proportional to the number of patients included. PBO: Placebo; NEMO60Q4W: Nemolizumab 60 mg every 4 weeks; BARI2: Baricitinib 2 mg daily; TRALO300Q2W: Tralokinumab 300 mg every other week; BARI4: Baricitinib 4 mg daily; ABRO100: Abrocitinib 100 mg daily; LEBRI250Q2W: Lebrikizumab 250 mg every other week; DUPI300Q2W: Dupilumab 300 mg every other week; UPA15: Upadacitinib 15 mg daily; ABRO200: Abrocitinib 200 mg daily; UPA30: Upadacitinib 30 mg daily. TEZE280Q2W: Tezepalumab 280 mg every other week. The numbers between the lines (1,2,3) indicate the number of studies comparing both treatments.

**Figure 3:**
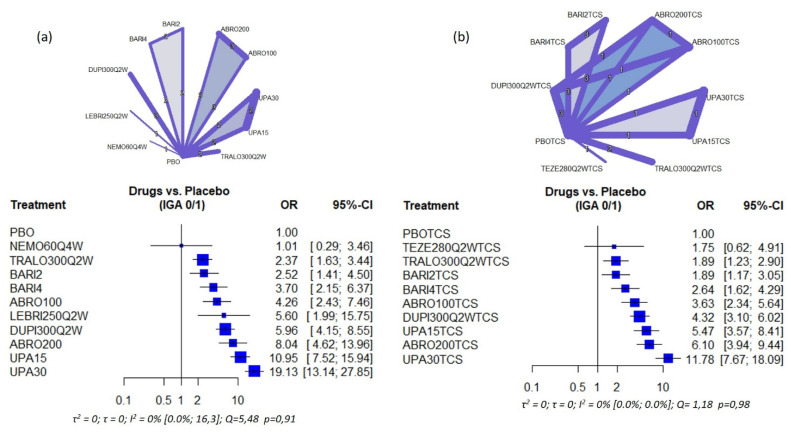
Network plot and Forest plot of the IGA 0/1 results obtained in the NMA (**a**) Monotherapy (**b**) Combination with TCS. The number in the lines that join each intervention represents the number of studies comparing both drugs; the thickness of the line is proportional to the number of patients included PBO: Placebo; NEMO60Q4W: Nemolizumab 60 mg every 4 weeks; TRALO300Q2W: Tralokinumab 300 mg every other week; BARI2: Baricitinib 2 mg daily; BARI4: Baricitinib 4 mg daily; ABRO100: Abrocitinib 100 mg daily; LEBRI250Q2W: Lebrikizumab 250 mg every other week; DUPI300Q2W: Dupilumab 300 mg every other week; ABRO200: Abrocitinib 200 mg daily; UPA15: Upadacitinib 15 mg daily; UPA30: Upadacitinib 30 mg daily. TEZE280Q2W: Tezepalumab 280 mg every other week. The numbers between the lines (1,2,3) indicate the number of studies comparing both treatments.

**Figure 4:**
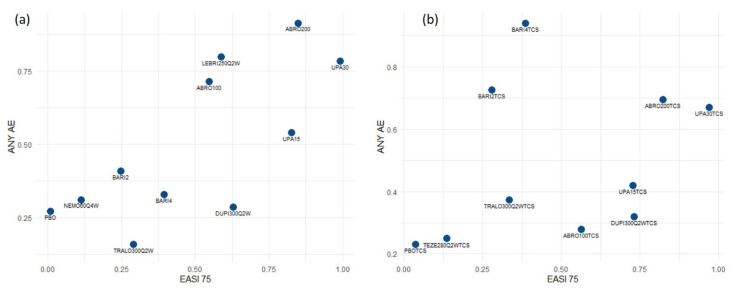
Comparison of efficacy and safety parameters. SUCRA EASI 75 and any AE. The drugs in the upper right quadrant are the most effective but with the most adverse effects. (**a**) Monotherapy (**b**) Combination with TCS. ABRO100: Abrocitinib 100 mg daily; ABRO200: Abrocitinib 200 mg daily; BARI2: Baricitinib 2 mg daily; BARI4: Baricitinib 4 mg daily; DUPI300Q2W: Dupilumab 300 mg every other week; LEBRI250Q2W: Lebrikizumab 250 mg every other week; NEMO60Q4W: Nemolizumab 60 mg every 4 weeks; PBO: Placebo; TEZE280Q2W: Tezepalumab 280 mg every other week; TRALO300Q2W: Tralokinumab 300 mg every other week; UPA15: Upadacitinib 15 mg daily; UPA30: Upadacitinib 30 mg daily.

The geometry of the different network plots for the different variables and analysis groups is shown in Figure 2 and Figure 3, as well as in Figure 3 and Figure 4. For both the variables EASI 75 and IGA 0/1, in monotherapy, a star geometry can be observed, with 14 studies; 11 treatments, 55 possible pairwise comparisons, and 13 pairwise comparisons with direct data (28 pairwise direct comparisons in studies). In the network with association with TCS, 9 studies were observed; 10 treatments, 45 possible pairwise comparisons, and 14 pairwise comparisons with direct data (22 pairwise direct comparisons in studies) (Figure 2 and Figure 3)

Considering all the studies evaluated:

- The evaluation period was considered as the primary endpoint to assess the efficacy of the molecule ranged between 12–16 weeks.
- Baseline mean EASI and Severity Scoring Atopic Dermatitis (SCORAD) were 32.22 and 65.33 respectively. 43.68% of the patients presented IGA 4 and the mean baseline NRS itch was 7.21.
- The mean duration of the disease was 25.24 years (Range: 17.7–38 years).
- The trials included 56.58% of men (range) with a mean age of 36.62 years-old (30–46 range). Most of the patients were Caucasians (66.83%).

### 3.2. Risk of Bias Assessment

Figure S1 shows the risk of bias graph and the risk of bias summary. Some risks of bias have been found in the different dimensions considered. A Dupilumab study showed a domain with high risk (and 3 unclear) of the total of 7 domains analyzed [11], as well as a nemolizumab study (and 1 unclear) [33], both in the “performance bias” domain. “Between studies” (Figure S1b), other dimensions more affected were “Allocation concealment” and “Detection bias”, showing 20–30% of “unclear” rating.

On the other hand, for the funnel plots performed for the different variables EASI75 and IGA 0/1, in monotherapy and combination with TCS, some asymmetries have been found (for example, Dupilumab 300 mg Q2W in Figure S2a). There are studies from which data could not be obtained.

### 3.3. Efficacy

#### 3.3.1. Efficacy of Direct Comparisons of Drug versus Placebo (with or without TCS Associated)

Treatment response was evaluated in all of them by determining the EASI75. The rest of the efficacy parameters (EASI50,90,100 and IGA0/1) were not evaluated in all studies. Quality of life measurement using DLQI was only used in 11 studies.

All the drugs evaluated showed superior efficacy when compared to placebo for all efficacy outcomes, both in the primary objectives of our study (EASI75/90), and secondary (IGA0/1 and NRSP). Nevertheless, Nemolizumab 60 mg Q4W in monotherapy did not reach statistical significance in the improvement of EASI 75/90 or IGA 0/1, with values of the confidence interval for OR that includes 1. In fact, nemolizumab only reaches statistical significance for NRSP. Tepezelumab in combination with TCS also did not reach statistical significance Figure 2, Figure 3, Figures S3 and S4).

Regarding Baricitinib, the efficacy was always lower to Abrocitinib 100 and 200 mg daily, Upadacitinib 15 and 30 mg daily and Dupilumab 300 mg/2 weeks in all of the measured parameters (EASI75, EASI90, and IGA0/1) with the 2 mg and 4 mg dose.

#### 3.3.2. Ranking of Treatments by Efficacy

(a) Primary objectives: In those studies where the drug was evaluated against placebo, a higher percentage of patients with EASI75 was obtained with Upadacitinib 30 mg [OR: 18.90 (13.94; 25.62)] followed by Abrocitinib 200 mg [OR = 11.26 (7.02; 18.05)] and Upadacitinib 15 mg [OR: 10.89 (8.13; 14.59)]. These results were also replicated in studies where the use of TCS was allowed (OR = 9.43; 6.12; 5.20, respectively) (Figure 2).

In the EASI90 comparation, the differences between the two networks, drugs alone or when TCS is allowed, are important (Figure S4). In both cases, the most effective drug in this parameter is Upadacitinib 30 mg daily. However, if we compare the ORs with respect to dupilumab, the differences are much greater in monotherapy [OR = 23.06 (19.90–33.46) vs. OR = 6.45 (4.5–9.55)] than in combination with TCS [OR = 11.22 (7.46–16.86) vs. OR = 5.57 (3.67–8.46)].

(b) Secondary objectives: About IGA0/1, however, the percentage of patients who reached it was higher with both doses of Upadacitinib 30 mg [OR: 19.13 (13.14; 27.85)] and 15 mg [OR = 10.95 (7.52; 15.94). In cases where the use of TCS was allowed, however, the dose of Abrocitinib 200 mg [OR = 6.10 (3.94; 9.44)] showed similar efficacy than Upadacitinib 15 mg [OR = 5.47 (3.57; 8.41)] (Figure 3).

In those studies where the quality of life was determined using DLQI, improvement achieved was always greater than placebo with statistically significant differences (with and without TCS). NRS pruritus showed a significantly lower reduction with all drugs evaluated (with and without TCS) (Figures S3 and S4).

### 3.4. Safety

Regarding the safety of the drugs evaluated in this systematic review,

(a) The risk of presenting any adverse effect, in the studies where the drug was evaluated on monotherapy, was higher in the case of Upadacitinib 30 mg versus placebo [OR: 1.64 (1.23; 2.18)] and both doses of Abrocitinib versus placebo, both 100 mg [OR: 1.56 (1.02; 2.38)] as 200 mg [OR: 2.06 [1.34; 3.17)]. If the concomitant use of TCS was allowed, OR was statistically significant for the dose of Baricitinib, 4 mg (OR: 2.36 [1.63; 3.42]) and 2 mg [OR = 1.72 (1.22; 2.41)], respectively (Figure S5a,b).

(b) If we focus on Severe Adverse Events (SAE) in those studies on monotherapy, no drug showed an OR higher than placebo. Furthermore, the 2 and 4 mg doses of Baricitinib presented a significantly lower vs placebo with statistically significant differences (Figure S5c,d).

Of all the studies analyzed, and considering upper respiratory tract infections, only the use of Upadacitinib 30 mg on monotherapy showed an OR higher than placebo with a statistically significant difference (OR = 1.79 (1.14; 2.82)].

In order to compare efficacy and safety, Figure 4 shows the surface under the cumulative ranking curve (SUCRA) of EASI 75 and any AE. Right upper quadrant drugs are the most effective ones, although they show a higher propensity to generate adverse events.

### 3.5. Heterogeneity Study

The analysis of heterogeneity showed great homogeneity between the different studies with I^2^ values of 0 or close to 0, and *p* > 0.1 in all the variables analyzed. For example, in the case of EASI75, the I^2^ value was 0 [0.0%; 18.4%], *p* = 0.90 in the monotherapy network; and 0 [0.0%; 59.2%], *p* = 0.64 in combination with TCS.

### 3.6. Consistency Analysis

Table S3 shows the analysis performed using net splitting. This method splits our network estimates the contribution of direct and indirect evidence. The RoR column shows the ratio of direct versus indirect ratios. No value reached *p* value < 0.05, which implies that there is a significant disagreement (inconsistency) between the direct and indirect estimate. However, there are some values to take into account, such as baricitinib in EASI 75 monotherapy (RoR 66.53 in the comparison Baricitinig 4 mg vs placebo) and abrocitinib in IGA0/1 monotherapy (RoR 576261.05 in the comparison Abrocitinib 200 mg vs. placebo).

## 4. Discussion

This meta-analysis is based on 23 RCTs, including 14 studies evaluating the drug versus placebo and 9 in which concomitant use of TCS was allowed. In these studies, 6780 patients were included, including monotherapy placebo-controlled clinical trials, and 3905 patients in those in which the use of concomitant TCS was allowed. This is the first NMA in which monotherapy and combination with TCS results are presented as differentiated. Janus kinase inhibitors Upadacitinib, Abrocitinib, and Dupilumab provided clinically meaningful effectiveness both in EASI75 and IGA 0/1. JAK inhibitors show higher efficacy than Dupilumab, but indeed a higher risk of adverse effects. These data confirm previous results reported in literature [37,38].

Most of the included trials have maintained uniformity regarding the inclusion criteria (with the exception already mentioned of including in some adolescent cases), although there are some protocols that required the lack of control of cyclosporine [31]. Thus, we can observe that the baseline characteristics of the different studies show a similar population in severity, age, time of evolution of AD, etc. However, variability and poor documentation of inclusion criteria and baseline severity assessments in RCT for AD has been described [39]. This could contribute in part to the differences found in this NMA. The measurement of the efficacy variables also follows homogeneity in included studies, although some studies have had to be excluded from the quantitative study because they express the results in numerical reduction instead of response rates.

Reviews of RCTs of AD drugs have been presented in two groups: those based on monotherapy, and those in which the concomitant use of TCS was allowed, based in “routine clinical practice”. In routine clinical practice, combination with TCS and emollients is common. Therefore, perhaps NMA in combination with TCS can better reflect the results of daily practice. Furthermore, both in monotherapy RCTs and in combination with TCS, the obligation by protocol to administer emollients is common. The importance that vehicles can have in the RCT design has been described, although practically no article is well defined [40]. However, combination therapy studies present an added difficulty to be interpreted, not being easy to decipher which of the results are due to the active drug and which to the TCS. For this reason, the results obtained in both modalities with the same drug should not be assessed jointly. Our meta-analysis divided studies into two different nets. Several studies have shown relatively high efficacy for placebo arm so the effect of the studied drug (Lebrikizumab [18,32], Baricitinib [20,27], Nemolizumab [34]) may look diminished, perhaps due to the use of emollients, as we have commented before. Contrarily, the efficacy of other molecules such as Upadacitinib shows almost no improvement with the addition of TCS [36]. All these facts result in important differences between our two NMA networks. The addition of TCS causes a decrease in the effectiveness difference between Upadacitinib, Abrocitinib, and Dupilumab. Although Upadacitinib 30 mg daily has been considered the most effective drug in achieving EASI75, the OR when compared to placebo is 18.9 in monotherapy, and slightly higher than 9 in combination with TCS. Although there are statistical techniques that allow estimating the effect of each treatment in combined RCT arms [41], they require some link in the NMA networks that does not exist at present between RCTs of AD in adults. Indeed, we have two isolated NMA networks, one where exclusively monotherapy drugs studies are analyzed, and the other one where both the active drug and the comparator arms are associated with TCS, so it is not possible to apply these techniques.

Some risks of bias have been found, especially in the “performance bias” domain, where a study of Dupilumab [11] and another of nemolizumab [33] showed a high risk of bias. Other domains with 25–30% unclear risk were “Allocation concealment” and “detection bias”. It must be taken into account that there are differences between the number of RCTs and patients included in this NMA. Thus, for example, molecules such as Lebrikizumab, Nemolizumab, and Tezepelumab present only one clinical trial in only one of the networks. The funnel chart is a tool to detect publication biases. We have found certain asymmetries and studies from which data could not be obtained, which is why a publication bias is possible that must be taken into account in the possible underestimation of the results.

The RCTs included in the present study are those that measure the primary endpoint at week 12–16. However, considering that AD is a chronic disease, long-term studies are interesting. Some studies suggest that when longer periods of time (such as 52 weeks or more) are considered, other results can be obtained. This may be due to the faster action of JAK inhibitors. In the decisions about which drug to administer, certain subjective variables such as quality of life can be viewed differently according to the different stakeholders involved (patients, doctors, health providers). In this NMA, variables such as DLQI have not been measured due to being measured differently, or not being measured.

We have analyzed the safety in terms of adverse effects, severe adverse effects, upper tract infections, and withdrawals. Dupilumab and Tralokilumab showed the lowest risk of adverse effects in monotherapy. However, in combination with TCS, Dupilumab and Abrocitinib 100 mg showed the lowest risk. This incidence is lower compared with the Janus kinase inhibitors Upadacitinib and Abrocitinib 200 mg. The comparison of efficacy and safety of different therapeutic options will help dermatology specialists to choose the most convenient option, based on patients’ characteristics. However, it is important to remember that our NMA collects short-term studies in a relatively small number of patients. Given that these drugs are designed for long-term use, the practical scope of these findings may be limited.

### Limitations

This meta-analysis shows some limitations. We have included drugs and RCTs arms with future potential to be used in atopic dermatitis. This led us to include molecules still in the development phase in our protocol. For those drugs that still do not have phase 3 RCT results, the doses tested in phase 2 have been chosen, so some differences on the final approved dose may be found. In addition, we have included all the studies completed and presented at conferences and with data available in Trialsgov^®^ but not published yet. This implies that some drugs have only some phase 2 RCTs with few patients. However, as our protocol was defined, it was necessary to include them. The analysis excluding studies with less than 50 subjects per arm hardly modifies the results.

Although our objective was to analyze the efficacy and safety of drugs in the adult population, the development of several molecules had included a small number of patients from 12 to 17 years of age, without being able to differentiate the results from the adult population. Disaggregated data on adolescents have not been presented yet, therefore it is not possible to perform a sub-analysis or omit these patients. For those drugs that have had different development for the adolescent and the adult population, the results have been similar [11,42]. For this reason, we decided to include those studies and analyze the studies that included both adolescent and adult populations.

Finally, we have not carried out the quality of the evidence study, which would have facilitated the interpretation of the results and decision-making.

## 5. Conclusions

In summary, with the existing evidence, the new JAK inhibitors (Upadacitinib and Abrocitinib), at higher doses, are the most effective drugs for the short-term treatment of moderate-to-severe atopic dermatitis. However, these doses showed the highest risk for any adverse event. Furthermore, the concomitant use of TCS modifies the ranking and ORs. All this, together with the great heterogeneity and complexity of atopic dermatitis, makes it difficult to transfer general recommendations.

However, our results may provide a useful basis for the preparation of treatment guidelines for the use of new generation of therapies in moderate to severe atopic dermatitis.

## Figures and Tables

**Table 1:** Characteristics of the studies and baseline population included in the systematic review.

Publication Data				Study Arm Design			Study Arm Baseline Characteristics									
Study	Study ID	Year	Phase	Agent ID	Agent, Dosing, Schedule, Route	n	Males (%)	Age (Mean)	Adolescent (12–17 Years) (%)	Race (White %)	Disease Duration (Year, Mean)	Basal EASI Score (Mean)	BSA Score (Mean)	Basal SCORAD (Mean)	Basal NRS Itch (Mean)	IGA 4 (%)
1	SIMPSON SOLO-1 [11]	2016	3	Placebo	sc	224	53	39.0	0	65	28.0	31.8	57.0	67.0	7.7	49
			3	Dupilumab	300 mg Q2W sc	224	58	38.0	0	69	26.0	30.4	53.4	65.1	7.6	48
			3	Dupilumab	300 mg Q1W sc	223	64	39.0	0	67	26.0	29.8	54.5	65.9	7.7	48
2	SIMPSON SOLO-2 [11]	2016	3	Placebo	Sc	236	56	35.0	0	66	26.0	30.5	53.3	68.9	7.7	49
			3	Dupilumab	300 mg Q2W sc	233	59	34.0	0	71	24.5	28.6	50.0	67.8	7.8	49
			3	Dupilumab	300 mg Q1W sc	239	58	35.0	0	70	24.0	29.0	50.0	67.4	7.8	47
3	THAÇI [12]	2016	2b	Dupilumab	300 mg QW sc	63	68	36.2	0	NR	27.9	30.1	48.4	65.0	6.54	49
			2b	Dupilumab	300 mg Q2W sc	64	64	39.4	0	NR	30.5	33.8	53.2	68.5	6.74	47
			2b	Placebo	sc	61	66	37.2	0	NR	29.8	32.9	51.1	67.1	6.34	48
			2b	Dupilumab	200 Q2W sc	61	59	35.8	0	NR	25.2	32.9	50.8	68.3	6.98	49
			2b	Dupilumab	300 Q4W sc	65	62	36.8	0	NR	26.5	29.4	50.8	67.2	6.84	43
			2b	Dupilumab	100 Q4W sc	65	52	36.3	0	NR	27.9	32.2	48.7	68.2	6.71	48
4	BLAUVELT CHRONOS [13]	2017	3	Placebo + TCS	sc	315	61	34.0	0	66	26.0	29.6	55.0	64.1	7.6	47
			3	Dupilumab + TCS	300 mg Q2W sc	106	58	40.5	0	70	28.0	30.9	58.8	69.7	7.7	50
			3	Dupilumab + TCS	300 mg QW sc	319	60	34.0	0	65	26.0	29.0	52.0	65.3	7.4	46
5	RUZICKA [14]	2017	2	placebo		53	47	37.0	NR	NR	NR	29.0	44.8	NR	7.5	49.0
			2	Nemolizumab	0.1 mg/kg Q4W sc	53	53	33.5	NR	NR	NR	32.4	55.9	NR	7.5	58.4
			2	Nemolizumab	0.5 mg/kg Q4W sc	54	41	33.7	NR	NR	NR	28.6	45.5	NR	7.6	55.6
			2	Nemolizumab	2.0 mg/kg Q4W sc	52	60	34.4	NR	NR	NR	28.2	48.8	NR	7.6	53.9
			2	Nemolizumab	2.0 mg/kg Q8W sc	52	56	35.8	NR	NR	NR	29.0	45.8	NR	7.8	51.9
6	BRUIN-WELLER CAFÉ [15]	2018	3	Placebo + TCS	sc	108	63.0	37.5	0	96.3	28.5	31.7	53.0	67.5	6.9	48.1
			3	Dupilumab + TCS	300 mg Q2W sc	107	60.7	38.0	0	97.2	29.0	31.6	55.0	66.7	7.0	46.7
			3	Dupilumab + TCS	300 mg QW sc	110	60.0	38.0	0	95.5	32.0	31.1	55.8	66.1	6.4	47.3
7	SIMPSON [16]	2018	2	Placebo + TCS	sc	56	53.6	38.8	0	75.0	NR	24.9	NR	58.66	7.62	17.9
			2	Tezepelumab + TCS	280Q2W sc	55	58.2	38.6	0	59.1	NR	24.1	NR	57.68	7.76	18.2
8	WOLLENBERG [17]	2018	2b	Placebo + TCS	sc	51	43.1	39.4	0	60.8	NR	26.4	NR	58.5	NR	39.2
			2b	Tralokinumab + TCS	45Q2W sc	50	58.0	39.1	0	66.0	NR	24.8	NR	57.5	NR	36.0
			2b	Tralokinumab + TCS	150Q2W sc	51	51.0	37.1	0	64.7	NR	27.1	NR	60.8	NR	39.2
			2b	Tralokinumab + TCS	300Q2W sc	52	63.5	35.7	0	53.8	NR	27.3	NR	60.8	NR	44.2
9	SIMPSON [18]	2018	2	Lebrikizumab	125 mg SD sc	52	65.4	34.9	0	69	NR	24.6	44.2	56.5	NR	NR
			2	Lebrikizumab	250 mg SD sc	53	58.5	34.4	0	81	NR	26.3	50.5	58.9	NR	NR
			2	Lebrikizumab	125 mg Q4W sc	51	68.6	36.6	0	71	NR	26.9	48.5	60.8	NR	NR
			2	Placebo	Sc	53	67.9	38.7	0	66	NR	23.6	43.4	59.2	NR	NR
10	GUTTMAN [19]	2018	2b	Placebo + TCS	PO	49	49	35	0	47	17.7	22.1	NR	55	7	NR
			2b	Baricitinib + TCS	2 mg QD PO	37	59	42	0	54	26.4	22.1	NR	53.3	6	NR
			2b	Baricitinib + TCS	4 mg QD PO	38	58	32.5	0	47	22.0	19.5	NR	57.6	6.5	NR
11	SIMPSON BREEZE-AD-1 [20]	2019	3	Placebo		249	59.4	35	0	59.5	26	32	53	68	6.7	42.2
			3	Baricitinib	1 mg QD PO	127	61.4	36	0	58.3	27	29	47	66	6.1	41.7
			3	Baricitinib	2 mg QD PO	123	66.7	35	0	61.0	25	31	50	68	6.4	42.3
			3	Baricitinib	4 mg QD PO	125	66.4	37	0	56.5	25	32	52	68	6.5	40.8
12	SIMPSON BREEZE-AD-2 [20]	2019	3	Placebo	PO	244	63.1	35	0	69.3	25	33	52	68	6.8	49.6
			3	Baricitinib	1 mg Q1D PO	125	64.0	33	0	68.0	24	33	55	67	6.4	50.8
			3	Baricitinib	2 mg QD PO	123	52.8	36	0	69.1	24	35	55	69	6.6	50.4
			3	Baricitinib	4 mg QD PO	123	66.7	34	0	66.7	23	33	54	68	6.6	51.2
13	GOODERHAM [21]	2019	2b	Placebo	PO	56	37.5	42.6	0	71.4	25.6	25.4	40.1	65.0	7.6	38.2
			2b	Abrocitinib	100 mg QD PO	56	55.4	41.1	0	71.4	23.8	26.7	41.9	65.4	7.4	47.3
			2b	Abrocitinib	200 mg QD PO	55	50.9	38.7	0	67.3	19.6	24.6	38.0	62.7	6.9	37.0
14	SIMPSON [22]	2019	2	Placebo	PO	64	39.1	37.7	0	NR	24.2	24.0	39.6	NR	NR	28.1
			2	Apremilast	30 mg QD PO	58	53.4	39.2	0	NR	24.1	24.2	42.0	NR	NR	31.0
			2	Apremilast	40 mg QD PO	63	49.2	38.3	0	NR	25.0	23.6	43.2	NR	NR	19.0
15	BLAUVELT [23]	2019	2	Dupilumab	300 mg QW sc	97	51	39	0	62	28	29	46	NR	7.4	NR
			2	Placebo	sc	97	47	40	0	69	27	31	49	NR	7.3	NR
16	GUTTMAN [24]	2019	2	Placebo	sc	27	51.9	43	0	74.1	38	34	57	64	8	51.9
			2	Dupilumab	200 mg QW sc	27	59.3	35	0	70.4	25	30	43	65	8	48.1
17	WOLLENBERG ECZTRA-1 [25]	2020	3	Placebo	sc	199	61.8	37.0	0	69.3	28.0	30.3	52.5	70.8	7.9	51.3
			3	Tralokinumab	300 mg Q2W sc	603	58.2	37.0	0	70.6	27.0	28.2	50.0	69.2	7.9	50.6
18	WOLLENBERG ECZTRA-2 [25]	2020	3	Placebo	sc	201	56.7	30.0	0	61.2	25.0	29.6	50.0	69.9	8.1	50.2
			3	Tralokinumab	300 mg Q2W sc	593	60.5	34.0	0	63.1	25.5	28.2	50.0	69.5	8.0	48.2
19	SILVERBERG ECZTRA-3 [26]	2020	3	Placebo + TCS	sc	127	66.1	34.0	0	66.9	26.0	26.5	40.0	67.9	8.0	47.2
			3	Tralokinumab + TCS	300 mg Q2W sc	253	49.4	37.0	0	80.2	27.0	24.7	41.0	66.2	8.0	45.8
20	REICH BREEZE-AD-7 [27]	2020	3	Placebo + TCS	PO	109	62	33.7	0	46	22.0	28.5	48.1	66.6	6.8	44
			3	Baricitinib + TCS	2 mg QD PO	109	61	33.8	0	50	24.6	29.3	50.6	66.8	6.3	46
			3	Baricitinib + TCS	4 mg QD PO	111	64	33.9	0	54	25.5	30.9	52.1	68.3	6.0	45
21	SIMPSON JADE-MONO-1 [28]	2020	3	Placebo		77	64	31.5	22	81	22.5	28.7	47.4	64.5	7.0	40
			3	Abrocitinib	100 mg QD PO	156	58	32.6	22	72	24.9	31.3	50.8	67.1	6.9	41
			3	Abrocitinib	200 mg QD PO	154	53	33.0	21	68	22.7	30.6	49.9	64.3	7.1	41
22	SILVERBERG JADE-MONO-2 [29]	2020	3	Placebo	PO	78	60.3	33.4	10.3	51.3	21.7	28.0	48.2	64.3	6.7	33.3
			3	Abrocitinib	100 mg QD PO	158	59.5	37.4	10.8	63.9	21.1	28.4	48.7	63.8	7.1	32.3
			3	Abrocitinib	200 mg QD PO	155	56.8	33.5	9.7	58.7	20.5	29.0	47.7	64.1	7.0	31.6
23	BIEBER JADE COMPARE [30]	2020	3	Placebo + TCS	PO	131	58.8	37.4	0	66.4	21.4	31.0	48.9	NR	7.1	32.8
			3	Abrocitinib + TCS	100 mg QD PO	238	50.4	37.3	0	76.5	22.7	30.3	48.1	NR	7.1	35.7
			3	Abrocitinib + TCS	200 mg QD PO	226	46.0	38.8	0	71.2	23.4	32.1	50.8	NR	7.6	38.9
			3	Dupilumab + TCS	300 mg Q2W sc	242	44.6	37.1	0	72.7	22.8	30.4	46.5	NR	7.3	33.1
24	BIERBER BREEZE-AD-4 [31]	2020	3	Placebo + TCS	PO	93	52.7	38.7	0	79.6	NR	309	48.4	69.1	7.1	53.8
			3	Baricitinib + TCS	1 mg QD PO	93	62.4	38.9	0	75.3	NR	34.3	56.6	70.9	6.7	50.5
			3	Baricitinib + TCS	2 mg QD PO	185	71.9	37.3	0	78.4	NR	30.6	50.1	67.8	6.7	50.5
			3	Baricitinib + TCS	4 mg QD PO	92	62.0	38.7	0	77.2	NR	32.7	53.9	68.2	6.7	51.1
25	GUTTMAN [32]	2020	2b	Placebo	sc	52	53.8	42.2	0	50.0	24.4	28.9	46.5	NR	7.4	38.5
			2b	Lebrikizumab	125 Q4W sc	73	37.0	36.7	0	50.7	22.8	29.9	45.5	NR	7.6	41.1
			2b	Lebrikizumab	250 Q4W sc	80	41.2	40.2	0	52.5	23.3	26.2	41.1	NR	7.1	32.5
			2b	Lebrikizumab	250 Q2W sc	75	34.7	38.9	0	53.3	22.1	25.5	39.4	NR	7.6	29.3
26	KABASHIMA [33]	2020	3	Nemolizumab	60 mg Q4W sc	143	65	39.0	4.9	NR	30.3	24.2	NR	NR	7.57	43
			3	Placebo	sc	72	67	40.5	5.6	NR	28.9	22.7	NR	NR	7.51	38
27	SILVERBERG [34]	2020	2b	Placebo + TCS	sc	57	54.4	40.9	0	78.9	NR	NR	45.6	NR	8.16	33.3
			2b	Nemolizumab + TCS	10 mg Q4W sc	55	52.7	35.3	0	69.1	NR	NR	40.4	NR	8.62	32.7
			2b	Nemolizumab + TCS	30 mg Q4W sc	57	50.9	40.2	0	70.2	NR	NR	42.4	NR	7.52	31.6
			2b	Nemolizumab + TCS	90 mg Q4W sc	57	45.6	40.9	0	77.2	NR	NR	37.6	NR	8.01	35.1
28	GUTTMAN MEASURE UP-1 [35]	2020	3	Upadacitinib	15 mg QD PO	281	55.9	34.1	14.9	NR	NR	30.6	48.5	NR	7.2	45.2
			3	Upadacitinib	30 mg QD PO	285	54.6	33.6	14.7	NR	NR	29.0	47.0	NR	7.3	46.0
			3	Placebo	PO	281	51.2	34.4	14.2	NR	NR	28.8	45.7	NR	7.3	44.5
29	GUTTMAN MEASURE UP-2 [35]	2020	3	Upadacitinib	15 mg QD PO	276	56.2	33.3	12.0	NR	NR	28.6	45.1	NR	7.2	54.3
			3	Upadacitinib	30 mg QD PO	282	57.4	34.1	12.4	NR	NR	29.7	47.0	NR	7.3	55.3
			3	Placebo	PO	278	55.4	33.4	12.9	NR	NR	29.1	47.6	NR	7.3	55.0
30	REICH MEASURE AD Up [36]	2020	3	Placebo + TCS	PO	304	58.6	34.3	13.2	NR	24.3	30.3	48.6	NR	7.1	53.6
			3	Upadacitinib + TCS	15 mg QD PO	300	59.7	32.5	13.0	NR	22.9	29.2	46.7	NR	7.1	52.3
			3	Upadacitinib + TCS	30 mg QD PO	297	64.0	35.5	12.5	NR	23.1	29.7	48.5	NR	7.4	52.9

NR: not reported. sc: subcutaneous. PO: Per Oral. Q2W: every other week; QW: every week; Q4W: every 4 weeks; SD: Single dose; QD: once a day. The duration of all the studies is 16 weeks, except for Jade-Mono 1 [28] and 2 [29] and Jade-Compare [30], where the primary objective is 12 weeks. 7 studies [14,17,18,22,23,24,34] were excluded from the quantitative analysis (you can see the reasons for exclusion in Table S4).

## Supplementary Materials

The following are available online at https://www.mdpi.com/article/10.3390/life11090927/s1, Figure S1: Forest plot of the results of EASI 50 and 90 and NRS pruritus 0/1 obtained in the NMA in Monotherapy; Figure S2: Forest plot of the results of EASI 50 and 90 and NRS pruritus 0/1 obtained in the NMA in combination with TCS; Figure S3: Funnel Plot obtained in the NMA (a) EASI 75 monotherapy; (b) EASI 75 in combination with TCS; (c) IGA 0/1 monotherapy; (d) IGA 0/1 in combination with TCS; Figure S4: Analysis of risk of bias. Figure S5: Forest plot of safety results. (a) At least one adverse effect in monotherapy; (b) At least one adverse effect in combination with TCS; (c) At least one severe adverse effect in monotherapy; (d) At least one severe adverse effect in combination with TCS. Table S1: Data extracted from the studies included in the NMA. Table S2: SUCRA and probability of each drug for the different variables studied in the NMA. Table S3: Separate indirect from direct evidence (SIDE) using back-calculation method. Table S4. Studies excluded in the quantitative analysis, with the cause of exclusion ScriptR: R-project program script used to run the meta analysis.
