# Supplementary material for: Short-Term Effectiveness and Safety of Biologics and Small Molecule Drugs for Moderate to Severe Atopic Dermatitis: A Systematic Review and Network Meta-Analysis
Source: Life (Basel). 2021 Sep 6;11(9):927. doi: 10.3390/life11090927 (PMC8470048; doi:10.3390/life11090927)
Supplement: Supplementary file 1 [file life-11-00927-s001.zip › Supplementary Materials.pdf]

life

MDPI

*Systematic Review*

# Short-Term Effectiveness and Safety of Biologics and Small Molecule Drugs for Moderate to Severe Atopic Dermatitis: A Systematic Review and Network Meta-Analysis

**José-Juan Pereyra-Rodriguez [1,2,*], Sara Alcantara-Luna [3], Javier Domínguez-Cruz [1], Manuel Galán-Gutiérrez [4], Ricardo Ruiz-Villaverde [5], Samuel Vilar-Palomo [6] and Jose-Carlos Armario-Hita [7]**

## Supplementary Materials

**Figure S1.** Analysis of risk of bias . a) Graph of risk of bias   b) Summary of risk of bias ("between the studies"). The risk of bias analysis focused on the primary objectives of the systematic review (EASI 75/90).

**Figure S2.** Funnel Plot obtained in the NMA a) EASI 75 monotherapy; b) EASI 75 in combination with TCS; c) IGA 0/1 monotherapy; d) IGA 0/1 in combination with TCS.

**Figure S3.** Forest plot of the results of EASI 50 and 90 and NRS pruritus 0/1 obtained in the NMA in Monotherapy. ABRO100: Abrocitinib 100 mg daily; ABRO200: Abrocitinib 200 mg daily; BARI2: Baricitinib 2 mg daily; BARI4: Baricitinib 4 mg daily; DUPI300Q2W: Dupilumab 300 mg every other week; LEBRI250Q2W: Lebrokizumab 250 mg every other week;   NEMO60Q4W: Nemolizumab 60 mg every 4 weeks; PBO: Placebo; TRALO300Q2W: Tralokinumab 300 mg every other week; UPA15: Upadacitinib 15 mg daily; UPA30: Upadacitinib 30 mg daily.

**Figure S4.** Forest plot of the results of EASI 50 and 90 and NRS pruritus 0/1 obtained in the NMA in combination with TCS. BARI2: Baricitinib 2 mg daily; BARI4: Baricitinib 4 mg daily; DUPI300Q2W: Dupilumab 300 mg every other week; LEBRI250Q2W: Lebrokizumab 250 mg every other week;   NEMO60Q4W: Nemolizumab 60 mg every 4 weeks; PBO: Placebo; TEZE280Q2W: Tezepalumab 280 mg every other week; TRALO300Q2W: Tralokinumab 300 mg every other week; UPA15: Upadacitinib 15 mg daily; UPA30: Upadacitinib 30 mg daily.

**Figure S5.** Forest plot of safety results. a) At least one adverse effect in monotherapy; b) At least one adverse effect in combination with TCS; c) At least one severe adverse effect in monotherapy; d) At least one severe adverse effect in combination with TCS.   BARI2: Baricitinib 2 mg daily; BARI4: Baricitinib 4 mg daily; DUPI300Q2W: Dupilumab 300 mg every other week; LEBRI250Q2W: Lebrokizumab 250 mg every other week;   NEMO60Q4W: Nemolizumab 60 mg every 4 weeks; PBO: Placebo; TEZE280Q2W: Tezepalumab 280 mg every other week; TRALO300Q2W: Tralokinumab 300 mg every other week; UPA15: Upadacitinib 15 mg daily; UPA30: Upadacitinib 30 mg daily.

**Table S1.** Data extracted from the studies included in the NMA.

**Table S2.** SUCRA and probability of each drug for the different variables studied in the NMA.

**Table S3.** Separate indirect from direct evidence (SIDE) using back-calculation method.

**Table S4.** Supplementary material eTable 4. Studies excluded in the quantitative analysis, with the cause of exclusión.

**Scheme. criptR**: R-project program script used to run the meta análisis (TXT).

**Records excluded after reading full text (Excel).**

**PRISMA NMA checklist (Word).**
