# Supplementary material for: Short-Term Effectiveness and Safety of Biologics and Small Molecule Drugs for Moderate to Severe Atopic Dermatitis: A Systematic Review and Network Meta-Analysis
Source: Life (Basel). 2021 Sep 6;11(9):927. doi: 10.3390/life11090927 (PMC8470048; doi:10.3390/life11090927)
Supplement: Supplementary file 1 [file life-11-00927-s001.zip › TableS1.pdf]

Supplementary material Table S1. Data extracted from the studies included in the NMA

Study #	StudyID	AgentID	n	EASI 50 (n)	EASI 75 (n)	EASI 90 (n)	EASI 100	Adjusted Mean DLQI (SD)	IGA =0/1	4-point improvement itch semana16ʃ	At leat one AE	At least one serious AE	At least one infectious AE	Upper respiratory tract	MACEs	Withdrawal due to AE
1	SIMPSON SOLO-1[1]	Placebo	224	55	33	17	NR	-5.3(0.5)	23	26/212	145	11	63	5	NR	2
		Dupilumab 300 mg Q2W	224	154	115	80	NR	-9.3(0.4)	85	87/213	167	7	80	6	NR	4
		Dupilumab 300 mg Q1W	223	136	117	74	NR	-9.0(0.4)	83	81/201	150	2	74	11	NR	4
2	SIMPSON SOLO-2[1]	Placebo	236	52	28	17	NR	-3.6(0.5)	20	21/221	168	13	76	5	NR	5
		Dupilumab 300 mg Q2W	233	152	103	70	NR	-9.3(0.4)	84	81/225	154	4	65	7	NR	2
		Dupilumab 300 mg Q1W	239	146	115	73	NR	-9.5(0.4)	87	89/228	157	8	68	9	NR	3
3	THAÇI[2]	Dupilumab 300 mg QW	63	52	38[a]	23[a]	NR	NR	21	NR	53	1	NR	5	NR	1
		Dupilumab 300 mg Q2W	64	50	34	19	NR	NR	19	NR	50	2	NR	6	NR	4
		Placebo	61	18	7	1	NR	NR	1	NR	49	4	NR	11	NR	3
		Dupilumab 200 mg Q2W	61	38	34	19	NR	NR	17	NR	46	1	NR	2	NR	3
		Dupilumab 300 mg Q4W	65	46	32	19	NR	NR	14	NR	56	3	NR	5	NR	3
		Dupilumab 100 mg Q4W	65	29	19	10	NR	NR	8	NR	53	5	NR	5	NR	10
4	BLAUVELT CHRONOS[3]	Placebo+TCS	315	118	73	35	NR	-5.3(0.31)	39	59/299	214	6	NR	NR	NR	15
		Dupilumab 300 mg Q2W +TCS	106	85	73	42	NR	-9.7(0.51)	41	60/102	81	3	NR	NR	NR	1
		Dupilumab 300 mg QW +TCS	319	249	204	138	NR	-10.5(0.30)	125	150/295	227	4	NR	NR	NR	8
5	RUZICKA[4]	placebo	53	16	8	NR	NR	NR	NR	NR	36	1	NR	6	NR	1
		Nemolizumab 0.1mg/kg Q4W	53	50	15	NR	NR	NR	NR	NR	38	1	NR	4	NR	5
		Nemolizumab 0.5mg/kg Q4W	54	24	17	NR	NR	NR	NR	NR	36	0	NR	1	NR	3
		Nemolizumab 2.0mg/kg Q4W	52	21	10	NR	NR	NR	NR	NR	40	2	NR	4	NR	4
		Nemolizumab 2.0mg/kg Q8W	52	NR	NR	NR	NR	NR	NR	NR	37	2	NR	5	NR	3
6	BRUIN-WELLER CAFE[5]	Placebo+TCS	108	47	32	13	NR	-4.5(0.49)	15	13/91	75	2	44	1	NR	1
		Dupilumab 300 mg Q2W +TCS	107	91	67	49	NR	-9.5(0.46)	43	43/94	77	2	49	1	NR	0
		Dupilumab 300 mg QW +TCS	110	94	65	41	NR	-8.8(0.45)	43	38/94	76	2	47	3	NR	2
7	SIMPSON[6]	Placebo+TCS	56	30	11	NR	NR	NR	7	NR	40	3	NR	7	NR	4
		Tezepelumab 280Q2W +TCS	55	36	13	NR	NR	NR	11	NR	38	2	NR	1	NR	2
8	WOLLENBERG[7]	Placebo+TCS	51	LOCF	LOCF	NR	NR	NR	6	NR	31	1	NR	5	NR	5
		Tralokinumab 45Q2W +TCS	50	LOCF	LOCF	NR	NR	NR	6	NR	36	3	NR	5	NR	2
		Tralokinumab 150Q2W +TCS	51	LOCF	LOCF	NR	NR	NR	10	NR	35	2	NR	5	NR	3
		Tralokinumab 300Q2W +TCS	52	LOCF	LOCF	NR	NR	NR	13	NR	30	0	NR	4	NR	0
9	SIMPSON[8]	Lebrikizumab 125 mg SD	52	36	20	NR	NR	NR	11	NR	38	3	24	NR	NR	1
		Lebrikizumab 250 mg SD	53	37	26	NR	NR	NR	15	NR	39	0	20	NR	NR	0
		Lebrikizumab 125 mg Q4W	51	42	28	NR	NR	NR	17	NR	27	2	12	NR	NR	2
		Placebo	53	33	18	NR	NR	NR	10	NR	35	2	24	NR	NR	1

10	GUTTMAN[9]	Placebo+TCS	49	LOCF	10	3	NR	NR	4	NR	24	0	NR	1	NR	5
		Baricitinib 2 mg QD +TCS	37	LOCF	11	7	NR	NR	8	NR	17	0	NR	0	NR	1
		Baricitinib 4 mg QD +TCS	38	LOCF	13	8	NR	NR	8	NR	27	1	NR	2	NR	5
11	SIMPSON BREEZE-AD-1[10]	Placebo	249	NR	22	12	NR	NR	12	6	135	6	NR	6	0	4
		Baricitinib 1 mg QD	127	NR	22	11	NR	NR	15	10	69	1	NR	1	0	2
		Baricitinib 2 mg QD	123	NR	23	13	NR	NR	14	12	71	0	NR	3	0	1
		Baricitinib 4 mg QD	125	NR	31	20	NR	NR	21	24	73	2	NR	4	0	1
12	SIMPSON BREEZE-AD-2[10]	Placebo	244	NR	15	6	NR	NR	11	10	137	9	NR	5	0	2
		Baricitinib 1 mg QD	125	NR	16	8	NR	NR	11	6	66	9	NR	6	0	7
		Baricitinib 2 mg QD	123	NR	22	11	NR	NR	13	16	71	3	NR	5	0	3
		Baricitinib 4 mg QD	123	NR	26	16	NR	NR	17	20	66	1	NR	4	0	2
13	GOODERHAM[11]	Placebo	56	14	8	5	NR	NR	3	13	NR	NR	13	5	NR	NR
		Abrocitinib 100 mg QD	56	30	22	14	NR	NR	16	25	NR	NR	24	3	NR	NR
		Abrocitinib 200 mg QD	55	38	31	21	NR	NR	21	28	NR	NR	23	5	NR	NR
14	SIMPSON[12]	Placebo	64	NR	NR	NR	NR	NR	NR	NR	30	0	NR	8	NR	1
		Apremilast 30mg QD	58	NR	NR	NR	NR	NR	NR	NR	36	1	NR	2	NR	2
		Apremilast 40mg QD	63	NR	NR	NR	NR	NR	NR	NR	44	2	NR	3	NR	6
15	BLAUVELT[13]	Dupilumab 300mg QW	97	70	52	NR	NR	NR	43	NR	54	3	NR	11	NR	5
		Placebo	97	31	19	NR	NR	NR	10	NR	60	0	NR	14	NR	0
16	GUTTMAN[14]	Placebo	27	6	4	0	NR	NR	0	NR	23	3	NR	4	NR	4
		Dupilumab 200 mg QW	27	21	18	9	NR	NR	10	NR	24	0	NR	4	NR	3
17	WOLLENBERG ECZTRA-1[15]	Placebo	199	42	25	8	NR	-5.0(0.59)	14	20/194	151	8	NR	2	NR	8
		Tralokinumab 300 mg Q2W	603	250	150	87	NR	-7.1(0.31)	95	119/595	460	23	NR	9	NR	20
18	WOLLENBERG ECZTRA-2[15]	Placebo	201	41	23	11	NR	-4.9(0.60)	22	19/200	132	5	NR	17	NR	3
		Tralokinumab 300 mg Q2W	593	295	196	108	NR	-8.8(0.30)	131	144/575	364	10	NR	59	NR	9
19	SILVERBERG ECZTRA-3[16]	Placebo+TCS	127	73	45	27	NR	-8.8(0.56)/	33	43/126	84	4	NR	6	NR	1
		Tralokinumab 300 mg Q2W +TCS	253	200	141	83	NR	-11.7(0.39)	98	113/249	180	2	NR	19	NR	6
20	REICH BREEZE-AD-7[17]	Placebo+TCS	109	45	25	15	NR	-5.6(0.6)	16	21/104	41	4	26	2	0	1
		Baricitinib 2 mg QD +TCS	109	70	47	18	NR	-7.5(0.6)	26	37/97	61	2	41	8	0	0
		Baricitinib4 mg QD +TCS	111	78	53	27	NR	-8.9(0.6)	34	44/100	64	4	37	3	0	5
21	SIMPSON JADE-MONO-1[18]	Placebo	77	17	9	4/76	NR	-4.2(-5.9 to-2.5)/60	6	11/74	44	3	NR	5	NR	7
		Abrocitinib 100 mg QD	156	90	62	29/156	NR	-7.0(-8.1 to-5.8)/121	37	55/147	108	5	NR	11	NR	9
		Abrocitinib 200 mg QD	154	116	96	59/153	NR	-9.1(-10.3 to-8.0)/119	67	84/147	120	5	NR	11	NR	9
22	SILVERBERG JADE-MONO-2[19]	Placebo	78	15	8	3/77	NR	-3.9(-5.3 to-2.4)/70	7	9/76	42	1	NR	3	NR	10
		Abrocitinib 100 mg QD	158	106	69	37/155	NR	-8.3(-9.3 to-7.3)/140	44	71/156	99	5	NR	14	NR	6
		Abrocitinib 200 mg QD	155	123	94	58/154	NR	-9.8(-10.7 to-8.8)/139	59	85/153	102	2	NR	5	NR	5
23	BIEBER JADE COMPARE 2021[20]	Placebo+TCS	131	NR	40	NR	NR	NR	17	NR	70	5	NR	NR	NR	5
		Abrocitinib 100 mg QD +TCS	238	NR	144	NR	NR	NR	83	NR	121	6	NR	NR	NR	6
		Abrocitinib 200 mg QD +TCS	226	NR	160	NR	NR	NR	107	NR	140	2	NR	NR	NR	10
		Dupilumab 300 mg Q2W+TCS	242	NR	159	NR	NR	NR	94	NR	121	2	NR	NR	NR	8
24	BIERBER BREEZE-AD-4[21]	Placebo+TCS	93	33	16	6	NR	-5.0(0.8)	9	7/85	50	2	NR	NR	0	1

		Baricitinib 1 mg QD +TCS	93	42	21	8	NR	-6.2(0.7)	12	18/78	58	4	NR	NR	0	0
		Baricitinib 2 mg QD +TCS	185	95	52	19	NR	-6.6(0.5)	28	38/166	125	3	NR	NR	0	3
		Baricitinib 4 mg QD +TCS	92	48	29	13	NR	-8.0(0.7)	20	29/76	69	6	NR	NR	0	1
25	GUTTMAN[22]	Placebo	52	24	8	3	NR	NR	5	6/22	24	2	NR	3	NR	1
		Lebrikizumab 125 Q4W	73	41	27	16	NR	NR	16	23/55	42	2	NR	6	NR	2
		Lebrikizumab 250 Q4W	80	50	38	25	NR	NR	22	27/57	39	0	NR	9	NR	4
		Lebrikizumab 250 Q2W	75	49	38	28	NR	NR	28	35/50	46	2	NR	2	NR	3
26	KABASHIMA[23]	Nemolizumab 60 mg Q4W	143	74	37	10	NR	NR	8	46	101	3	NR	NR	NR	3
		Placebo	72	29	13	3	NR	NR	4	9	51	2	NR	NR	NR	0
27	SILVERBERG[24]	Placebo+TCS	57	NR	NR	NR	NR	NR	NR	NR	43	0	23	NR	NR	0
		Nemolizumab 10mgQ4W +TCS	55	NR	NR	NR	NR	NR	NR	NR	47	1	34	NR	NR	3
		Nemolizumab 30mgQ4W +TCS	57	NR	NR	NR	NR	NR	NR	NR	47	2	34	NR	NR	2
		Nemolizumab 90mgQ4W +TCS	57	NR	NR	NR	NR	NR	NR	NR	48	2	34	NR	NR	3
28	GUTTMAN MEASURE-1[25]	Upadacitinib 15 mg QD	281	NR	197	149	47	NR	135	144/275	176	6	NR	25	0	4
		Upadacitinib 30 mg QD	285	NR	227	188	77	NR	177	167/279	209	8	NR	38	0	11
		Placebo	281	NR	46	23	5	NR	24	32/270	166	8	NR	20	0	12
29	GUTTMAN MEASURE-2[25]	Upadacitinib 15 mg QD	276	NR	166	117	39	NR	107	113/269	166	5	NR	19	0	11
		Upadacitinib 30 mg QD	282	NR	206	165	53	NR	147	166/278	173	7	NR	17	0	7
		Placebo	278	NR	37	15	2	NR	13	24/267	146	8	NR	12	0	12
30	REICH MEASURE AD Up[26]	Placebo+TCS	304	NR	80	40	NR	NR	33	44/294	190	9	NR	22	NR	7
		Upadacitinib 15mg QD +TCS	300	NR	195	128	NR	NR	120	149/288	200	7	NR	21	NR	4
		Upadacitinib 30mg QD +TCS	297	NR	229	187	NR	NR	175	186/291	215	4	NR	23	NR	4

NR: Not reported; LOCF: Data published as Last observed carried forward; SD: Standard deviation; AE: adverse evento; MACE: major adverse cardiovascular events; Q2W: every other week; QW: every week; Q4W: every 4 weeks; TCS: Topical corticosteroids; SD: Single doce; QD: once a day

ʃ patients with a baseline peak score of at least 4 on the numerical rating scale for pruritus

[a]Data from EASI75 and EASI 90 were derived by graphical visualization (numerical values were not available)

7 studies [4,7,8,12,13,14,24] were excluded from the quantitative analysis (you can see the reasons for exclusion in Table S4)

REFERENCES

1. Simpson, EL; Bieber, T; Guttman-Yassky, E; Beck, LA; Blauvelt, A; Cork, MJ; Silverberg, JI; Deleuran, M; Kataoka, Y; Lacour, J-P; et al. Two Phase 3 Trials of Dupilumab versus Placebo in Atopic Dermatitis. N Engl J Med **2016**, 375, 2335–48.
2. Thaçi, D; Simpson, EL; Beck, LA; Bieber, T; Blauvelt, A; Papp, K; Soong, W; Worm, M; Szepietowski, JC; Sofen, H; et al. Efficacy and safety of dupilumab in adults with moderate-to-severe atopic dermatitis inadequately controlled by topical treatments: a randomised, placebo-controlled, dose-ranging phase 2b trial. Lancet **2016**, 387, 40–52.
3. Blauvelt, A; de Bruin-Weller, M; Gooderham, M; Cather, JC; Weisman, J; Pariser, D; Simpson, EL; Papp, KA; Hong, HC-H; Rubel, D; et al. Long-term management of moderate-to-severe atopic dermatitis with dupilumab and concomitant topical corticosteroids (LIBERTY AD CHRONOS): a 1-year, randomised, double-blinded, placebo-controlled, phase 3 trial. Lancet **2017**, 389, 2287–303.
4. Ruzicka, T; Hanifin, JM; Furue, M; Pulka, G; Mlynarczyk, I; Wollenberg, A; Galus, R; Etoh, T; Mihara, R; Yoshida, H; et al. Anti-Interleukin-31 Receptor A Antibody for Atopic Dermatitis. N Engl J Med **2017**, 376, 826-35.
5. De Bruin-Weller, M; Thaci, D; Smith, C; Radin, A; Zhang, R; Gadkari, A; Eckert, L; Hultsch, T; Pirozzi, G; Graham, NMH; et al. Dupilumab with concomitant topical corticosteroids in adult Patients with atopic dermatitis who are not adequately controlled with or are intolerant to cyclosporine a, or when this treatment is medically inadvisable: a placebo-controlled, randomized phase III clinical trial. Br J Dermatol. **2018,** 178, 1083-1101.
6. Simpson, EL; Parnes, JR; She, D; Crouch, S; Rees, W; Mo, M; Van der Merwe, R. Tezepelumab, an anti–thymic stromal lymphopoietin monoclonal antibody, in the treatment of moderate to severe atopic dermatitis: a randomized phase 2a clinical trial. J Am Acad Dermatol **2019**, 80, 1013-1021.

7. Wollenberg, A; Howell, MD; Guttman-Yassky, E; Silverberg, JI; Kell, C; Ranade, K; Moate, R; van der Merwe, E. Treatment of atopic dermatitis with tralokinumab, an anti–IL-13 mAb . J Allergy Clin Immunol **2019**, 143, 135-41.
8. Simpson, EL; Flohr ,C; Eichenfield, LF; Bieber, T; Sofen, H; Taïeb, A; Owen, R; Putnam, W; Castro, M; DeBusk, K; et al. Efficacy and safety of lebrikizumab (an anti-IL-13 monoclonal antibody) in adults with moderate-to-severe atopic dermatitis inadequately controlled by topical corticosteroids: A randomized, placebo-controlled phase II trial (TREBLE). J Am Acad Dermatol **2018**, 78, 863-871.e11.
9. Guttman-Yassky, E; Silverberg, JI; Nemoto, O; Forman, SB; Wilke, A; Prescilla, R; de la Peña, A; Nunes, FP; Janes, J; Gamalo, M; et al. Baricitinib in adult patients with moderate-to-severe atopic dermatitis: A phase 2 parallel, double-blinded, randomized placebo-controlled multiple-dose study. J Am Acad Dermatol **2019**, 80, 913-921.e9.
10. Simpson, EL; Lacour, J-P; Spelman, L; Galimberti, R; Eichenfield, LF; Bissonnette, R; King, BA; Thyssen, JP; Silverberg, JI; Bieber, T; et al. Baricitinib in Patients with Moderate-to-Severe Atopic Dermatitis and Inadequate Response to Topical Corticosteroids: results from Two Randomised Monotherapy Phase 3 Trials. Br J Dermatol , 183, 242-245.
11. Gooderham, MJ; Forman, SB; Bissonnette, R; Beebe, JS; Zhang, W; Banfield, C; Zhu, L; Papacharalambous, J; Vincent, MS; Peeva, E. Efficacy and Safety of Oral Janus Kinase 1 Inhibitor Abrocitinib for Patients With Atopic Dermatitis: A Phase 2 Randomized Clinical Trial. JAMA Dermatology **2019**, 155, 1371–9.
12. Simpson, EL; Imafuku, S; Poulin, Y; Ungar, B; Zhou, L; Malik, K; Wen, H-C; Xu, H; Estrada, YD; Peng, X; et al. A Phase 2 Randomized Trial of Apremilast in Patients with Atopic Dermatitis. J Invest Dermatol **2019**, 139, 1063-1072.
13. Blauvelt, A; Simpson, EL; Tyring, SK; Purcell, LA; Shumel, B; Petro, CD; Akinlade, B; Gadkari, A; Eckert, L; Graham, NMH; et al. Dupilumab does not affect correlates of vaccine-induced immunity: A randomized, placebo-controlled trial in adults with moderate-to-severe atopic dermatitis. J Am Acad Dermatol **2019**, 80, 158-167.e1.
14. Guttman-Yassky, E; Bissonnette, R; Ungar, B; Suarez-Fariñas, M; Ardeleanu, M; Esaki, H; Suprun, M; Estrada, Y; Xu, H; Peng, H; et. Dupilumab progressively improves systemic and cutaneous abnormalities in patients with atopic dermatitis. J Allergy Clin Immunol. **2019,** 143, 155-172
15. Wollenberg, A; Blauvelt, A; Guttman-Yassky, E; Worm, M; Lynde, C; Lacour, J-P; Spelman, L; Katoh, N; Saeki, H; Poulin, Y; et al. Tralokinumab for moderate-to-severe atopic dermatitis: results from two 52-week, randomized, double-blind, multicentre, placebo-controlled phase III trials (ECZTRA 1 and ECZTRA 2). Br J Dermatol **2021**, 184, 437-449.
16. Silverberg, JI; Toth, D; Bieber, T; Alexis, AF; Elewski, BE; Pink, AE; Hijnen, D; Jensen, TN; Bang, B; Olsen, CK; et al. Tralokinumab plus topical corticosteroids for the treatment of moderate-to-severe atopic dermatitis: results from the double-blind, randomized, multicentre, placebo-controlled phase III ECZTRA 3 trial. Br J Dermatol **2021**, 184, 450-463
17. Reich, K; Kabashima, K; Peris, K; Silverberg, JI; Eichenfield, LF; Bieber, T; Kaszuba, A; Kolodsick, J; Yang, FE; Gamalo, M; et al. Efficacy and Safety of Baricitinib Combined With Topical Corticosteroids for Treatment of Moderate to Severe Atopic Dermatitis: a Randomized Clinical Trial. JAMA Dermatology **2020**, 156, 1133,1143.
18. Simpson, EL; Sinclair, R; Forman, S; Wollenberg, A; Aschoff, R; Cork, M; Bieber, T; Thyssen, JP; Yosipovitch, G; Flohr, C; et al. Efficacy and safety of abrocitinib in adults and adolescents with moderate-to-severe atopic dermatitis (JADE MONO-1): a multicentre, double-blind, randomised, placebo-controlled, phase 3 trial. Lancet **2020**;396:255–66.
19. Silverberg, JI; Simpson, EL; Thyssen, JP; Gooderham, M; Chan, G; Feeney, C; Biswas, P; Valdez, H; Dibonaventura, M; Nduaka, C; et al. Efficacy and Safety of Abrocitinib in Patients with Moderate-to-Severe Atopic Dermatitis: a Randomized Clinical Trial. JAMA Dermatology **2020,** 156, 863-873.
20. Bieber, T; Simpson, EL; Silverberg, JI; Thaçi, D; Paul, C; Pink, AE; Kataoka, Y; Chu, C-Y; DiBonaventura, M; Rojo, R; et al. Abrocitinib versus Placebo or Dupilumab for Atopic Dermatitis. N Engl J Med **2021,** 384, 1101–12.
21. Bieber, T; Reich, K; Paul, C; Tsunemi, Y; Augustin, M; Lacour, J-P; Ghislain, P-D; Dutronc, Y; Liao, R; Gamalo, M; et al. Efficacy and Safety of Baricitinib in Combination With Topical Corticosteroids in Patients With Moderate-to-Severe Atopic Dermatitis Who Failed, Are Intolerant to, or Have Contraindication to Cyclosporine: Results From a Randomized, Placebo-Controlled, Ph. Eur. Acad. Dermatology Venereol. (EADV Virtual), **2020**. https://eadvvirtualcongress.org/ (accessed 29/07/2021)
22. Guttman-Yassky, E; Blauvelt, A; Eichenfield, LF; Paller, AS; Armstrong, AW; Drew, J; Gopalan, R; Simpson, EL. Efficacy and Safety of Lebrikizumab, a High-Affinity Interleukin 13 Inhibitor, in Adults with Moderate to Severe Atopic Dermatitis: a Phase 2b Randomized Clinical Trial. JAMA Dermatology **2020**, 156, 411-420.
23. Kabashima, K; Matsumura, T; Komazaki, H; Kawashima, M. Trial of Nemolizumab and Topical Agents for Atopic Dermatitis with Pruritus. N Engl J Med **2020**, 383, 141–50.
24. Silverberg, JI; Pinter, A; Pulka, G; Poulin, Y; Bouaziz, J-D; Wollenberg, A; Murrell, DF; Alexis, A; Lindsey, L; Ahmad, F; et al. Phase 2B randomized study of nemolizumab in adults with moderate-to-severe atopic dermatitis and severe pruritus. J Allergy Clin Immunol **2020**, 145, 173–82.
25. Guttman-Yassky, E; Teixeira, HD; Simpson, EL; Papp, KA; Pangan, AL; Blauvelt, A; Thaçi, D; Chu, CY; Hong, HC ho; Katoh, N; et al. Once-daily upadacitinib versus placebo in adolescents and adults with moderate-to-severe atopic dermatitis (Measure Up 1 and Measure Up 2): results from two replicate double-blind, randomised controlled phase 3 trials. Lancet **2021**, 397, 2151–68.
26. Reich, K; Teixeira, HD; de Bruin-Weller, M; Bieber, T; Soong, W; Kabashima, K; Werfel, T; Zeng, J; Huang, X; Hu, X; et al. Safety and efficacy of upadacitinib in combination with topical corticosteroids in adolescents and adults with moderate-to-severe atopic dermatitis (AD Up): results from a randomised, double-blind, placebo-controlled, phase 3 trial. Lancet **2021**, 397, 2169–81.
