# Supplementary material for: Short-Term Effectiveness and Safety of Biologics and Small Molecule Drugs for Moderate to Severe Atopic Dermatitis: A Systematic Review and Network Meta-Analysis
Source: Life (Basel). 2021 Sep 6;11(9):927. doi: 10.3390/life11090927 (PMC8470048; doi:10.3390/life11090927)
Supplement: Supplementary file 1 [file life-11-00927-s001.zip › TableS2.pdf]

Table S2. SUCRA and probability of each drug for the different variables studied in the NMA

a) Monotherapy

	EASI 75		IGA 0/1		EASI 90		NRS P		ANY AE		UPPER TRACT INFECTION		SEVERE AE		WITHDRAW	
	SUCRA	Prob Best	SUCRA	Prob Best	SUCRA	Prob Best	SUCRA	Prob Best	SUCRA	Prob AE	SUCRA	Prob AE	SUCRA	Prob AE	SUCRA	Prob AE
ABRO100	0.55	0.00	0.50	0.00	0.42	0.00	0.35	0.00	0.71	0.04	0.58	0.10	0.64	0.08	0.17	0.00
ABRO200	0.85	0.06	0.77	0.01	0.76	0.03	0.69	0.02	0.91	0.48	0.36	0.02	0.73	0.18	0.15	0.00
BARI2	0.25	0.00	0.27	0.00	0.22	0.00	0.35	0.00	0.41	0.00	0.63	0.19	0.11	0.00	0.62	0.01
BARI4	0.40	0.00	0.44	0.00	0.42	0.00	0.68	0.04	0.33	0.00	0.62	0.19	0.04	0.00	0.50	0.00
DUPI300Q2W	0.63	0.00	0.65	0.00	0.60	0.00	0.53	0.00	0.29	0.00	0.30	0.01	0.31	0.00	0.54	0.00
LEBRI250Q2W	0.59	0.01	0.63	0.04	0.75	0.15	0.65	0.15	0.80	0.37	0.17	0.05	0.61	0.21	0.74	0.03
NEMO60Q4W	0.12	0.00	0.09	0.00	0.17	0.00	0.32	0.01	0.31	0.02	.	.	0.73	0.34	0.99	0.96
PBO	0.01	0.00	0.05	0.00	0.02	0.00	0.00	0.00	0.27	0.00	0.35	0.00	0.63	0.01	0.58	0.00
TRALO300Q2W	0.29	0.00	0.25	0.00	0.35	0.00	0.18	0.00	0.16	0.00	0.55	0.08	0.74	0.14	0.51	0.00
UPA15	0.83	0.00	0.86	0.00	0.82	0.00	0.78	0.01	0.54	0.00	0.65	0.07	0.35	0.00	0.31	0.00
UPA30	0.99	0.93	0.99	0.95	0.98	0.81	0.97	0.79	0.78	0.09	0.80	0.28	0.61	0.05	0.41	0.00

b) Association with topical corticosteroids (TCS)

	EASI 75		IGA 0/1		EASI 90		NRS P		ANY AE		UPPER TRACT INFECTION		SEVERE AE		WITHDRAW	
	SUCRA	Prob Best	SUCRA	Prob Best	SUCRA	Prob Best	SUCRA	Prob Best	SUCRA	Prob AE	SUCRA	Prob AE	SUCRA	Prob AE	SUCRA	Prob AE
ABRO100TCS	0.57	0.01	0.55	0.00	.	.	.	.	0.28	0.00	.	.	0.60	0.01	0.36	0.00
ABRO200TCS	0.82	0.12	0.83	0.05	.	.	.	.	0.69	0.08	.	.	0.26	0.00	0.49	0.00
BARI2TCS	0.28	0.00	0.24	0.00	0.25	0.00	0.36	0.01	0.72	0.02	0.75	0.33	0.40	0.00	0.39	0.00
BARI4TCS	0.39	0.00	0.42	0.00	0.46	0.00	0.58	0.05	0.94	0.62	0.69	0.19	0.80	0.01	0.73	0.01
DUPI300Q2WTCS	0.73	0.02	0.66	0.00	0.77	0.06	0.68	0.09	0.32	0.00	0.51	0.24	0.43	0.00	0.29	0.00
NEMO30Q4WTCS	.	.	.	.	.	.	.	.	0.61	0.19	.	.	0.99	0.97	0.99	0.97
PBOTCS	0.04	0.00	0.02	0.00	0.02	0.00	0.03	0.00	0.23	0.00	0.46	0.01	0.61	0.00	0.59	0.00
TEZE280Q2WTCS	0.14	0.00	0.25	0.00	.	.	.	.	0.25	0.02	0.07	0.00	0.44	0.00	0.40	0.00
TRALO300Q2WTCS	0.34	0.00	0.26	0.00	0.31	0.00	0.20	0.01	0.37	0.01	0.55	0.09	0.14	0.00	0.38	0.00
UPA15TCS	0.73	0.02	0.77	0.01	0.71	0.02	0.71	0.07	0.42	0.01	0.46	0.06	0.50	0.00	0.44	0.00
UPA30TCS	0.97	0.83	0.99	0.94	0.98	0.92	0.93	0.77	0.67	0.06	0.51	0.08	0.32	0.00	0.44	0.00

ABRO100: Abrocitinib 100 mg daily; ABRO200: Abrocitinib 200 mg daily; BARI2: Baricitinib 2 mg daily; BARI4: Baricitinib 4 mg daily; DUPI300Q2W: Dupilumab 300 mg every other week; LEBRI250Q2W: Lebrikizumab 250 mg every other week; NEMO60Q4W: Nemolizumab 60 mg every 4 weeks; PBO: Placebo; TEZE280Q2W: Tezepalumab 280 mg every other week;

TRALO300Q2W: Tralokinumab 300 mg every other week; UPA15: Upadacitinib 15 mg daily; UPA30: Upadacitinib 30 mg daily.

SUCRA: surface under the cumulative ranking curve; prob: probability; NRSP; numerical rating scale for pruritus; AE: adverse event
