# Supplementary material for: Short-Term Effectiveness and Safety of Biologics and Small Molecule Drugs for Moderate to Severe Atopic Dermatitis: A Systematic Review and Network Meta-Analysis
Source: Life (Basel). 2021 Sep 6;11(9):927. doi: 10.3390/life11090927 (PMC8470048; doi:10.3390/life11090927)
Supplement: Supplementary file 1 [file life-11-00927-s001.zip › TableS3.pdf]

Table S3. Separate indirect from direct evidence (SIDE) using back-calculation method

a) ***EASI 75 monotherapy***

comparison	k	prop	nma	direct	indir.	RoR	z	p-value
ABRO100:ABRO200	3	1.00	0.46	0.46	.	.	.	.
ABRO100:BARI2	0	0.00	1.86	.	1.86	.	.	.
ABRO100:BARI4	0	0.00	1.40	.	1.40	.	.	.
ABRO100:DUPI300Q2W	0	0.00	0.82	.	0.82	.	.	.
ABRO100:LEBRI250Q2W	0	0.00	0.91	.	0.91	.	.	.
ABRO100:NEMO60Q4W	0	0.00	3.24	.	3.24	.	.	.
ABRO100:PBO	3	0.99	5.14	5.21	1.70	3.06	0.50	0.62
ABRO100:TRALO300Q2W	0	0.00	1.74	.	1.74	.	.	.
ABRO100:UPA15	0	0.00	0.47	.	0.47	.	.	.
ABRO100:UPA30	0	0.00	0.27	.	0.27	.	.	.
ABRO200:BARI2	0	0.00	4.08	.	4.08	.	.	.
ABRO200:BARI4	0	0.00	3.07	.	3.07	.	.	.
ABRO200:DUPI300Q2W	0	0.00	1.80	.	1.80	.	.	.
ABRO200:LEBRI250Q2W	0	0.00	1.99	.	1.99	.	.	.
ABRO200:NEMO60Q4W	0	0.00	7.11	.	7.11	.	.	.
ABRO200:PBO	3	0.99	11.26	11.32	6.76	1.67	0.22	0.83
ABRO200:TRALO300Q2W	0	0.00	3.82	.	3.82	.	.	.
ABRO200:UPA15	0	0.00	1.03	.	1.03	.	.	.
ABRO200:UPA30	0	0.00	0.60	.	0.60	.	.	.
BARI2:BARI4	2	1.00	0.75	0.75	.	.	.	.
BARI2:DUPI300Q2W	0	0.00	0.44	.	0.44	.	.	.
BARI2:LEBRI250Q2W	0	0.00	0.49	.	0.49	.	.	.
BARI2:NEMO60Q4W	0	0.00	1.74	.	1.74	.	.	.
BARI2:PBO	2	1.00	2.76	2.76	2.53	1.09	0.02	0.99
BARI2:TRALO300Q2W	0	0.00	0.94	.	0.94	.	.	.
BARI2:UPA15	0	0.00	0.25	.	0.25	.	.	.
BARI2:UPA30	0	0.00	0.15	.	0.15	.	.	.
BARI4:DUPI300Q2W	0	0.00	0.59	.	0.59	.	.	.
BARI4:LEBRI250Q2W	0	0.00	0.65	.	0.65	.	.	.
BARI4:NEMO60Q4W	0	0.00	2.32	.	2.32	.	.	.
BARI4:PBO	2	1.00	3.67	3.69	0.06	66.53	0.58	0.56
BARI4:TRALO300Q2W	0	0.00	1.24	.	1.24	.	.	.
BARI4:UPA15	0	0.00	0.34	.	0.34	.	.	.
BARI4:UPA30	0	0.00	0.19	.	0.19	.	.	.
DUPI300Q2W:LEBRI250Q2W	0	0.00	1.11	.	1.11	.	.	.
DUPI300Q2W:NEMO60Q4W	0	0.00	3.95	.	3.95	.	.	.
DUPI300Q2W:PBO	3	1.00	6.25	6.25	.	.	.	.
DUPI300Q2W:TRALO300Q2W	0	0.00	2.12	.	2.12	.	.	.
DUPI300Q2W:UPA15	0	0.00	0.57	.	0.57	.	.	.
DUPI300Q2W:UPA30	0	0.00	0.33	.	0.33	.	.	.
LEBRI250Q2W:NEMO60Q4W	0	0.00	3.57	.	3.57	.	.	.
LEBRI250Q2W:PBO	1	1.00	5.65	5.65	.	.	.	.
LEBRI250Q2W:TRALO300Q2W	0	0.00	1.91	.	1.91	.	.	.

LEBRI250Q2W:UPA15	0	0.00	0.52	.	0.52	.	.	.
LEBRI250Q2W:UPA30	0	0.00	0.30	.	0.30	.	.	.
NEMO60Q4W:PBO	1	1.00	1.58	1.58	.	.	.	.
NEMO60Q4W:TRALO300Q2W	0	0.00	0.54	.	0.54	.	.	.
NEMO60Q4W:UPA15	0	0.00	0.15	.	0.15	.	.	.
NEMO60Q4W:UPA30	0	0.00	0.08	.	0.08	.	.	.
TRALO300Q2W:PBO	2	1.00	2.95	2.95	.	.	.	.
UPA15:PBO	2	1.00	10.89	10.89	10.53	1.03	0.01	0.99
UPA30:PBO	2	1.00	18.90	18.81	77.16	0.24	-0.53	0.60
TRALO300Q2W:UPA15	0	0.00	0.27	.	0.27	.	.	.
TRALO300Q2W:UPA30	0	0.00	0.16	.	0.16	.	.	.
UPA15:UPA30	2	1.00	0.58	0.58	.	.	.	.

b) ***EASI 75 Combination with TCS***

comparison	k	prop	nma	direct	indir.	RoR	z	p-value
ABRO100TCS:ABRO200TCS	1	1.00	0.63	0.63	.	.	.	.
ABRO100TCS:BARI2TCS	0	0.00	1.83	.	1.83	.	.	.
ABRO100TCS:BARI4TCS	0	0.00	1.52	.	1.52	.	.	.
ABRO100TCS:DUPI300Q2WTCS	1	0.90	0.76	0.80	0.48	1.67	0.87	0.38
ABRO100TCS:PBOTCS	1	0.73	3.87	3.49	5.14	0.68	-0.87	0.38
ABRO100TCS:TEZE280Q2WTCS	0	0.00	3.06	.	3.06	.	.	.
ABRO100TCS:TRALO300Q2WTCS	0	0.00	1.69	.	1.69	.	.	.
ABRO100TCS:UPA15TCS	0	0.00	0.74	.	0.74	.	.	.
ABRO100TCS:UPA30TCS	0	0.00	0.41	.	0.41	.	.	.
ABRO200TCS:BARI2TCS	0	0.00	2.89	.	2.89	.	.	.
ABRO200TCS:BARI4TCS	0	0.00	2.40	.	2.40	.	.	.
ABRO200TCS:DUPI300Q2WTCS	1	0.91	1.20	1.27	0.72	1.76	0.87	0.38
ABRO200TCS:PBOTCS	1	0.75	6.12	5.52	8.37	0.66	-0.87	0.38
ABRO200TCS:TEZE280Q2WTCS	0	0.00	4.84	.	4.84	.	.	.
ABRO200TCS:TRALO300Q2WTCS	0	0.00	2.68	.	2.68	.	.	.
ABRO200TCS:UPA15TCS	0	0.00	1.18	.	1.18	.	.	.
ABRO200TCS:UPA30TCS	0	0.00	0.65	.	0.65	.	.	.
BARI2TCS:BARI4TCS	3	0.99	0.83	0.84	0.30	2.81	0.45	0.65
BARI2TCS:DUPI300Q2WTCS	0	0.00	0.41	.	0.41	.	.	.
BARI2TCS:PBOTCS	3	0.99	2.12	2.11	2.63	0.80	-0.12	0.90
BARI2TCS:TEZE280Q2WTCS	0	0.00	1.67	.	1.67	.	.	.
BARI2TCS:TRALO300Q2WTCS	0	0.00	0.93	.	0.93	.	.	.
BARI2TCS:UPA15TCS	0	0.00	0.41	.	0.41	.	.	.
BARI2TCS:UPA30TCS	0	0.00	0.22	.	0.22	.	.	.
BARI4TCS:DUPI300Q2WTCS	0	0.00	0.50	.	0.50	.	.	.
BARI4TCS:PBOTCS	3	1.00	2.55	2.56	0.54	4.75	0.26	0.79
BARI4TCS:TEZE280Q2WTCS	0	0.00	2.01	.	2.01	.	.	.
BARI4TCS:TRALO300Q2WTCS	0	0.00	1.12	.	1.12	.	.	.
BARI4TCS:UPA15TCS	0	0.00	0.49	.	0.49	.	.	.
BARI4TCS:UPA30TCS	0	0.00	0.27	.	0.27	.	.	.

DUPI300Q2WTCS:PBOTCS	3	1.00	5.10	5.10	.	.	.	.
DUPI300Q2WTCS:TEZE280Q2WTCS	0	0.00	4.03	.	4.03	.	.	.
DUPI300Q2WTCS:TRALO300Q2WTCS	0	0.00	2.23	.	2.23	.	.	.
DUPI300Q2WTCS:UPA15TCS	0	0.00	0.98	.	0.98	.	.	.
DUPI300Q2WTCS:UPA30TCS	0	0.00	0.54	.	0.54	.	.	.
TEZE280Q2WTCS:PBOTCS	1	1.00	1.27	1.27	.	.	.	.
TRALO300Q2WTCS:PBOTCS	1	1.00	2.29	2.29	.	.	.	.
UPA15TCS:PBOTCS	1	1.00	5.20	5.20	.	.	.	.
UPA30TCS:PBOTCS	1	1.00	9.43	9.43	.	.	.	.
TEZE280Q2WTCS:TRALO300Q2WTCS	0	0.00	0.55	.	0.55	.	.	.
TEZE280Q2WTCS:UPA15TCS	0	0.00	0.24	.	0.24	.	.	.
TEZE280Q2WTCS:UPA30TCS	0	0.00	0.13	.	0.13	.	.	.
TRALO300Q2WTCS:UPA15TCS	0	0.00	0.44	.	0.44	.	.	.
TRALO300Q2WTCS:UPA30TCS	0	0.00	0.24	.	0.24	.	.	.
UPA15TCS:UPA30TCS	1	1.00	0.55	0.55	.	.	.	.

a) ***IGA 0/1 monotherapy***

**comparison**	**k**	**prop**	**nma**	**direct**	**indir.**	**RoR**	**z**	**p-value**
ABRO100:ABRO200	3	1	0.53	0.53	.	.	.	.
ABRO100:BARI2	0	0	1.69	.	1.69	.	.	.
ABRO100:BARI4	0	0	1.15	.	1.15	.	.	.
ABRO100:DUPI300Q2W	0	0	0.71	.	0.71	.	.	.
ABRO100:LEBRI250Q2W	0	0	0.76	.	0.76	.	.	.
ABRO100:NEMO60Q4W	0	0	4.22	.	4.22	.	.	.
ABRO100:PBO	3	1	4.26	4.27	0.01	561.95	0.51	0.61
ABRO100:TRALO300Q2W	0	0	1.79	.	1.79	.	.	.
ABRO100:UPA15	0	0	0.39	.	0.39	.	.	.
ABRO100:UPA30	0	0	0.22	.	0.22	.	.	.
ABRO200:BARI2	0	0	3.19	.	3.19	.	.	.
ABRO200:BARI4	0	0	2.17	.	2.17	.	.	.
ABRO200:DUPI300Q2W	0	0	1.35	.	1.35	.	.	.
ABRO200:LEBRI250Q2W	0	0	1.43	.	1.43	.	.	.
ABRO200:NEMO60Q4W	0	0	7.98	.	7.98	.	.	.
ABRO200:PBO	3	1	8.04	7.94	576261.05	0.00	-1.28	0.20
ABRO200:TRALO300Q2W	0	0	3.39	.	3.39	.	.	.
ABRO200:UPA15	0	0	0.73	.	0.73	.	.	.
ABRO200:UPA30	0	0	0.42	.	0.42	.	.	.
BARI2:BARI4	2	1	0.68	0.68	.	.	.	.
BARI2:DUPI300Q2W	0	0	0.42	.	0.42	.	.	.
BARI2:LEBRI250Q2W	0	0	0.45	.	0.45	.	.	.
BARI2:NEMO60Q4W	0	0	2.50	.	2.50	.	.	.
BARI2:PBO	2	1	2.52	2.52	.	.	.	.
BARI2:TRALO300Q2W	0	0	1.06	.	1.06	.	.	.
BARI2:UPA15	0	0	0.23	.	0.23	.	.	.
BARI2:UPA30	0	0	0.13	.	0.13	.	.	.
BARI4:DUPI300Q2W	0	0	0.62	.	0.62	.	.	.

BARI4:LEBRI250Q2W	0	0	0.66	.	0.66	.	.	.
BARI4:NEMO60Q4W	0	0	3.67	.	3.67	.	.	.
BARI4:PBO	2	1	3.70	3.70	.	.	.	.
BARI4:TRALO300Q2W	0	0	1.56	.	1.56	.	.	.
BARI4:UPA15	0	0	0.34	.	0.34	.	.	.
BARI4:UPA30	0	0	0.19	.	0.19	.	.	.
DUPI300Q2W:LEBRI250Q2W	0	0	1.06	.	1.06	.	.	.
DUPI300Q2W:NEMO60Q4W	0	0	5.92	.	5.92	.	.	.
DUPI300Q2W:PBO	3	1	5.96	5.96	.	.	.	.
DUPI300Q2W:TRALO300Q2W	0	0	2.51	.	2.51	.	.	.
DUPI300Q2W:UPA15	0	0	0.54	.	0.54	.	.	.
DUPI300Q2W:UPA30	0	0	0.31	.	0.31	.	.	.
LEBRI250Q2W:NEMO60Q4W	0	0	5.56	.	5.56	.	.	.
LEBRI250Q2W:PBO	1	1	5.60	5.60	.	.	.	.
LEBRI250Q2W:TRALO300Q2W	0	0	2.36	.	2.36	.	.	.
LEBRI250Q2W:UPA15	0	0	0.51	.	0.51	.	.	.
LEBRI250Q2W:UPA30	0	0	0.29	.	0.29	.	.	.
NEMO60Q4W:PBO	1	1	1.01	1.01	.	.	.	.
NEMO60Q4W:TRALO300Q2W	0	0	0.42	.	0.42	.	.	.
NEMO60Q4W:UPA15	0	0	0.09	.	0.09	.	.	.
NEMO60Q4W:UPA30	0	0	0.05	.	0.05	.	.	.
TRALO300Q2W:PBO	2	1	2.37	2.37	.	.	.	.
UPA15:PBO	2	1	10.95	10.96	8.51	1.29	0.09	0.93
UPA30:PBO	2	0.99	19.13	19.23	8.94	2.15	0.32	0.75
TRALO300Q2W:UPA15	0	0	0.22	.	0.22	.	.	.
TRALO300Q2W:UPA30	0	0	0.12	.	0.12	.	.	.
UPA15:UPA30	2	1	0.57	0.57	.	.	.	.

d) ***IGA 0/1 Combination with TCS***

comparison	k	prop	nma	direct	indir.	RoR	z	p-value
ABRO100TCS:ABRO200TCS	1	1	0.60	0.60	.	.	.	.
ABRO100TCS:BARI2TCS	0	0	1.92	.	1.92	.	.	.
ABRO100TCS:BARI4TCS	0	0	1.38	.	1.38	.	.	.
ABRO100TCS:DUPI300Q2WTCS	1	0.93	0.84	0.84	0.81	1.04	0.06	0.95
ABRO100TCS:PBOTCS	1	0.59	3.63	3.59	3.69	0.97	-0.06	0.95
ABRO100TCS:TEZE280Q2WTCS	0	0	2.08	.	2.08	.	.	.
ABRO100TCS:TRALO300Q2WTCS	0	0	1.92	.	1.92	.	.	.
ABRO100TCS:UPA15TCS	0	0	0.66	.	0.66	.	.	.
ABRO100TCS:UPA30TCS	0	0	0.31	.	0.31	.	.	.
ABRO200TCS:BARI2TCS	0	0	3.23	.	3.23	.	.	.
ABRO200TCS:BARI4TCS	0	0	2.31	.	2.31	.	.	.
ABRO200TCS:DUPI300Q2WTCS	1	0.93	1.41	1.42	1.36	1.04	0.06	0.95
ABRO200TCS:PBOTCS	1	0.58	6.10	6.03	6.19	0.97	-0.06	0.95
ABRO200TCS:TEZE280Q2WTCS	0	0	3.48	.	3.48	.	.	.
ABRO200TCS:TRALO300Q2WTCS	0	0	3.23	.	3.23	.	.	.
ABRO200TCS:UPA15TCS	0	0	1.11	.	1.11	.	.	.

ABRO200TCS:UPA30TCS	0	0	0.52	.	0.52	.	.	.
BARI2TCS:BARI4TCS	3	1	0.72	0.72	0.43	1.66	0.15	0.88
BARI2TCS:DUPI300Q2WTCS	0	0	0.44	.	0.44	.	.	.
BARI2TCS:PBOTCS	3	0.99	1.89	1.90	0.97	1.96	0.25	0.80
BARI2TCS:TEZE280Q2WTCS	0	0	1.08	.	1.08	.	.	.
BARI2TCS:TRALO300Q2WTCS	0	0	1.00	.	1.00	.	.	.
BARI2TCS:UPA15TCS	0	0	0.34	.	0.34	.	.	.
BARI2TCS:UPA30TCS	0	0	0.16	.	0.16	.	.	.
BARI4TCS:DUPI300Q2WTCS	0	0	0.61	.	0.61	.	.	.
BARI4TCS:PBOTCS	3	1	2.64	2.63	.	.	.	.
BARI4TCS:TEZE280Q2WTCS	0	0	1.51	.	1.51	.	.	.
BARI4TCS:TRALO300Q2WTCS	0	0	1.40	.	1.40	.	.	.
BARI4TCS:UPA15TCS	0	0	0.48	.	0.48	.	.	.
BARI4TCS:UPA30TCS	0	0	0.22	.	0.22	.	.	.
DUPI300Q2WTCS:PBOTCS	3	1	4.32	4.32	.	.	.	.
DUPI300Q2WTCS:TEZE280Q2WTCS	0	0	2.47	.	2.47	.	.	.
DUPI300Q2WTCS:TRALO300Q2WTCS	0	0	2.29	.	2.29	.	.	.
DUPI300Q2WTCS:UPA15TCS	0	0	0.79	.	0.79	.	.	.
DUPI300Q2WTCS:UPA30TCS	0	0	0.37	.	0.37	.	.	.
TEZE280Q2WTCS:PBOTCS	1	1	1.75	1.75	.	.	.	.
TRALO300Q2WTCS:PBOTCS	2	1	1.89	1.89	.	.	.	.
UPA15TCS:PBOTCS	1	1	5.47	5.47	.	.	.	.
UPA30TCS:PBOTCS	1	1	11.78	11.78	.	.	.	.
TEZE280Q2WTCS:TRALO300Q2WTCS	0	0	0.93	.	0.93	.	.	.
TEZE280Q2WTCS:UPA15TCS	0	0	0.32	.	0.32	.	.	.
TEZE280Q2WTCS:UPA30TCS	0	0	0.15	.	0.15	.	.	.
TRALO300Q2WTCS:UPA15TCS	0	0	0.34	.	0.34	.	.	.
TRALO300Q2WTCS:UPA30TCS	0	0	0.16	.	0.16	.	.	.
UPA15TCS:UPA30TCS	1	1	0.46	0.46	.	.	.	.

K: Number of studies providing direct evidence; prop: Direct evidence proportion; nma Estimated treatment effect (OR) in network meta-analysis; direct:Estimated treatment effect (OR) derived from direct evidence; indir.: Estimated treatment effect (OR) derived from indirect evidence; RoR: Ratio of Ratios (direct versus indirect); z:z-value of test for disagreement (direct versus indirect); p-value:p-value of test for disagreement (direct versus indirect)

ABRO100: Abrocitinib 100 mg daily; ABRO200: Abrocitinib 200 mg daily; BARI2: Baricitinib 2 mg daily; BARI4: Baricitinib 4 mg daily; DUPI300Q2W: Dupilumab 300 mg every other week; LEBRI250Q2W: Lebrikizumab 250 mg every other week; NEMO60Q4W: Nemolizumab 60 mg every 4 weeks; PBO: Placebo; TEZE280Q2W: Tezepalumab 280 mg every other week; TRALO300Q2W: Tralokinumab 300 mg every other week; UPA15: Upadacitinib 15 mg daily; UPA30: Upadacitinib 30 mg daily. TCS: Topical corticosteroids
