# Supplementary material for: Short-Term Effectiveness and Safety of Biologics and Small Molecule Drugs for Moderate to Severe Atopic Dermatitis: A Systematic Review and Network Meta-Analysis
Source: Life (Basel). 2021 Sep 6;11(9):927. doi: 10.3390/life11090927 (PMC8470048; doi:10.3390/life11090927)
Supplement: Supplementary file 1 [file life-11-00927-s001.zip › TableS4.pdf]

Supplementary material Table S4. Studies excluded in the quantitative analysis, with the cause of exclusión

Studies excluded in the quantitative analysis, with the cause of exclusion	
RUZICKA 2017[1]	None of the doses of nemolizumab used have been used in phase III RCT.
WOLLENBERG 2018[2]	Data published as Last observed carried forward
SIMPSON 2018[3]	None of the doses of Lebrikizumab used have been used in phase III RCT.
SIMPSON 2019[4]	Data were reported as absolute reduction, not as EASI 75/90
BLAUVELT 2019[5]	None of the doses used are those approved for dupilumab
GUTTMAN 2019[6]	None of the doses used are those approved for dupilumab
SILVERBERG 2020[7]	Data were reported as absolute reduction, not as EASI 75/90

1. Egeberg A, Thyssen JP, Wu JJ, Ruzicka T, Hanifin JM, Furue M, et al. Anti-interleukin-31 receptor a antibody for atopic dermatitis. N Engl J Med 2017;376:2092.

2. Wollenberg A, Howell MD, Guttman-Yassky E, Silverberg JI, Birrell C, Kell C, et al. A Phase IIb dose-ranging efficacy and safety study of tralokinumab in adult patients with moderate-to-severe atopic dermatitis (AD). J Clin Aesthet Dermatol 2018;11:S13-.

3. Simpson EL, Flohr C, Eichenfield LF, Bieber T, Sofen H, Taïeb A, et al. Efficacy and safety of lebrikizumab (an anti-IL-13 monoclonal antibody) in adults with moderate-to-severe atopic dermatitis inadequately controlled by topical corticosteroids: A randomized, placebo-controlled phase II trial (TREBLE). J Am Acad Dermatol 2018;78:863-871.e11.

4. Simpson EL, Imafuku S, Poulin Y, Ungar B, Zhou L, Malik K, et al. A Phase 2 Randomized Trial of Apremilast in Patients with Atopic Dermatitis. J Invest Dermatol 2019.

5. Blauvelt A, Simpson EL, Tyring SK, Purcell LA, Shumel B, Petro CD, et al. Dupilumab does not affect correlates of vaccine-induced immunity: A randomized, placebo-controlled trial in adults with moderate-to-severe atopic dermatitis. J Am Acad Dermatol 2019;80:158-167.e1.

6. Guttman-Yassky E, Pangan AL, Silverberg JI, Thaci D, Hong CH, Mohamed MEF, et al. Primary results from a Phase IIb, randomized, placebo-controlled trial of upadacitinib for patients with atopic dermatitis. J Clin Aesthet Dermatol 2019;12:S16-S17.

7. Silverberg JI, Pinter A, Pulka G, Poulin Y, Bouaziz J-D, Wollenberg A, et al. Phase 2B randomized study of nemolizumab in adults with moderate-to-severe atopic dermatitis and severe pruritus. J Allergy Clin Immunol 2020;145:173–82.
