# Supplementary figures and images for: Short-Term Effectiveness and Safety of Biologics and Small Molecule Drugs for Moderate to Severe Atopic Dermatitis: A Systematic Review and Network Meta-Analysis
Source: Life (Basel). 2021 Sep 6;11(9):927. doi: 10.3390/life11090927 (PMC8470048; doi:10.3390/life11090927)

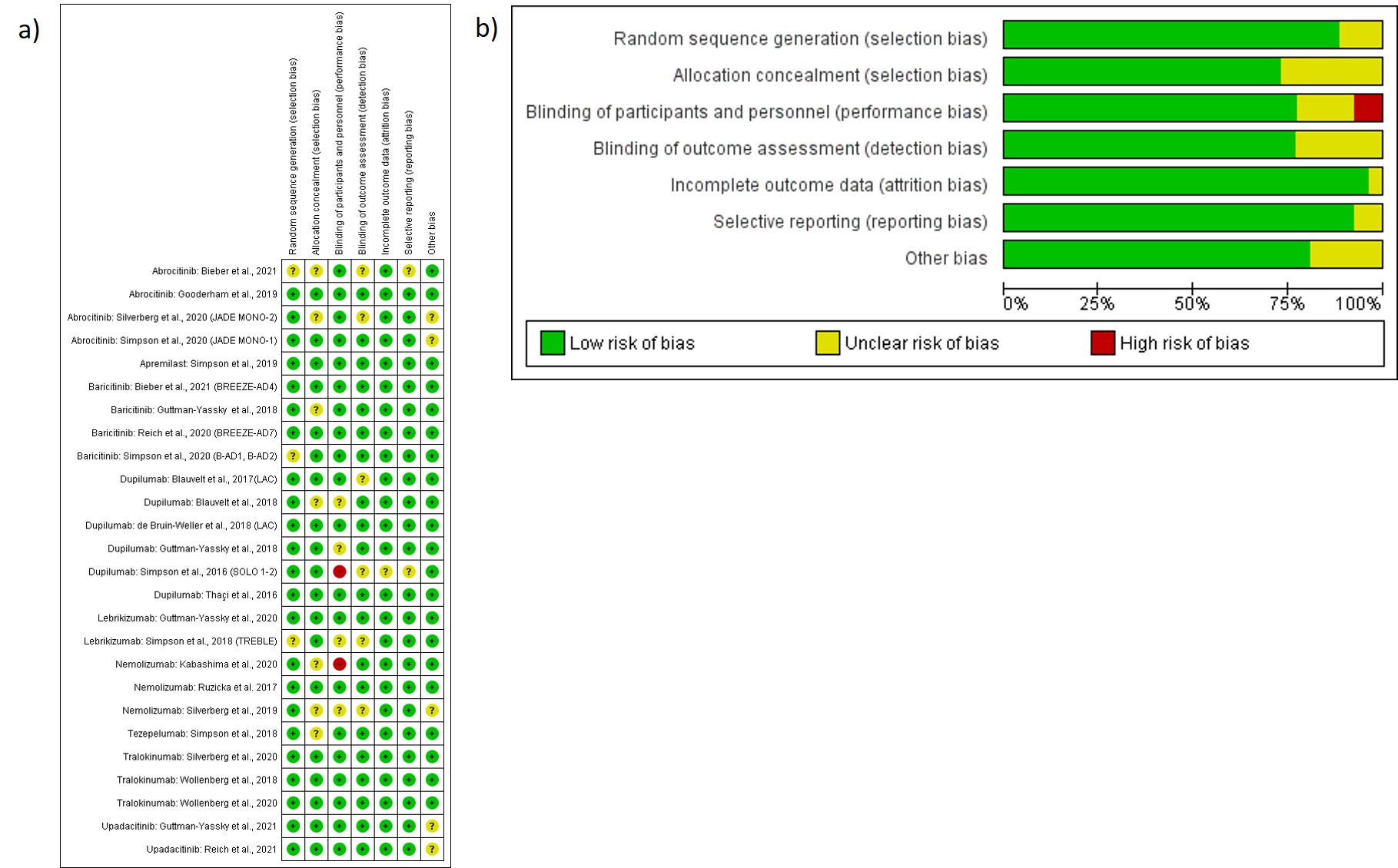

Supplement: Supplementary file 1 [file life-11-00927-s001.zip › FigureS1.jpg]

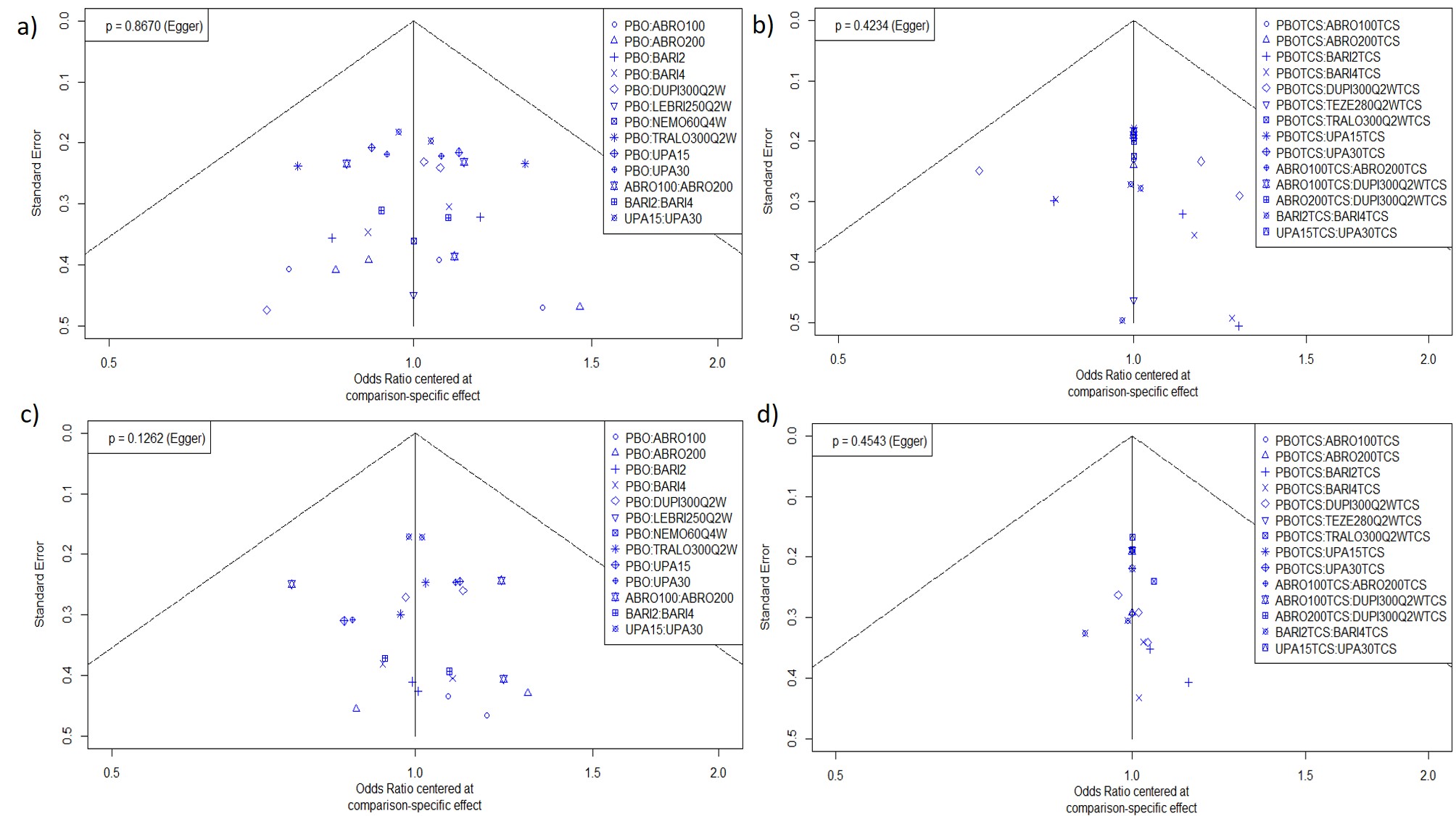

Supplement: Supplementary file 1 [file life-11-00927-s001.zip › FigureS2.jpg]

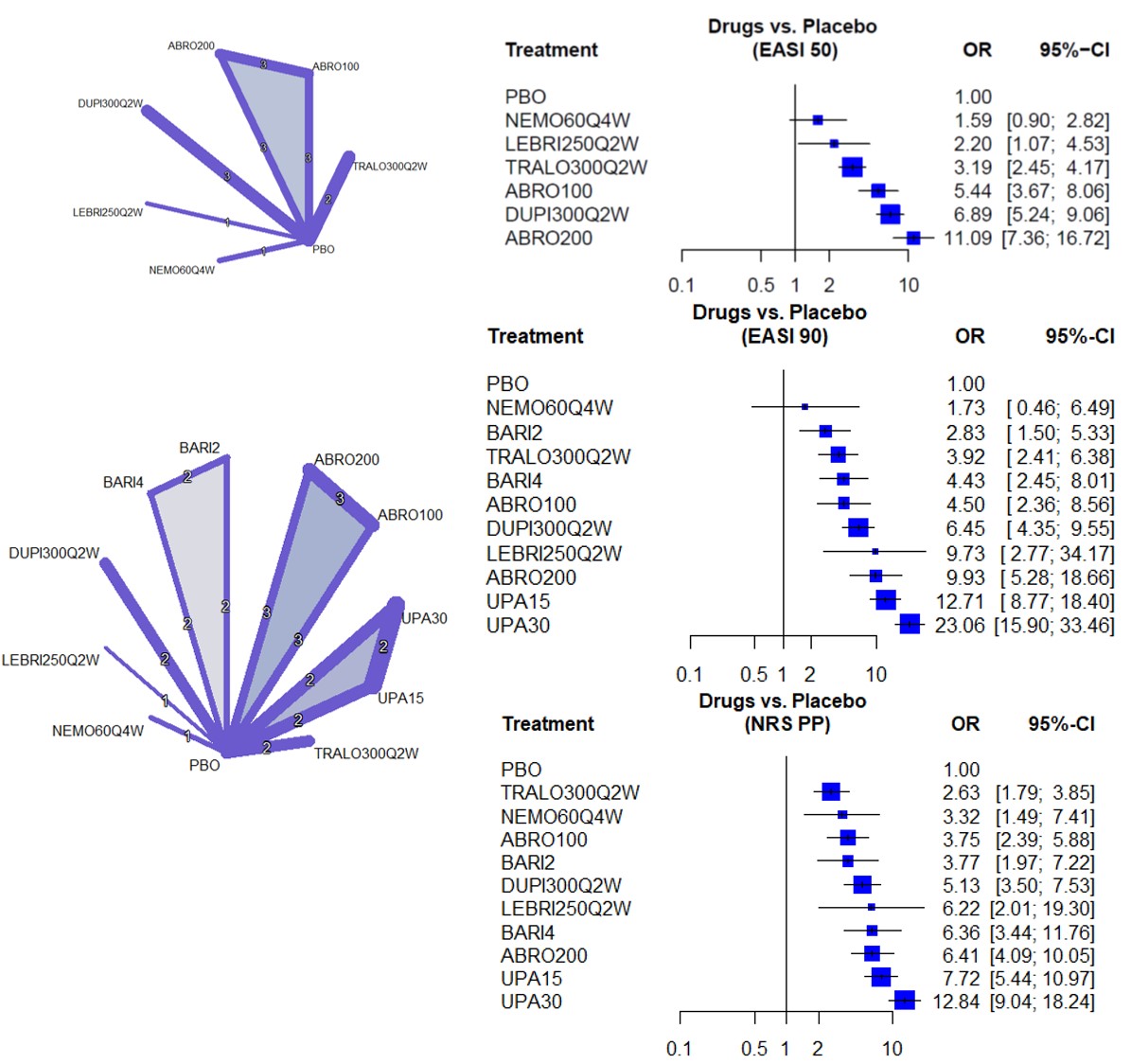

Supplement: Supplementary file 1 [file life-11-00927-s001.zip › FigureS3.jpg]

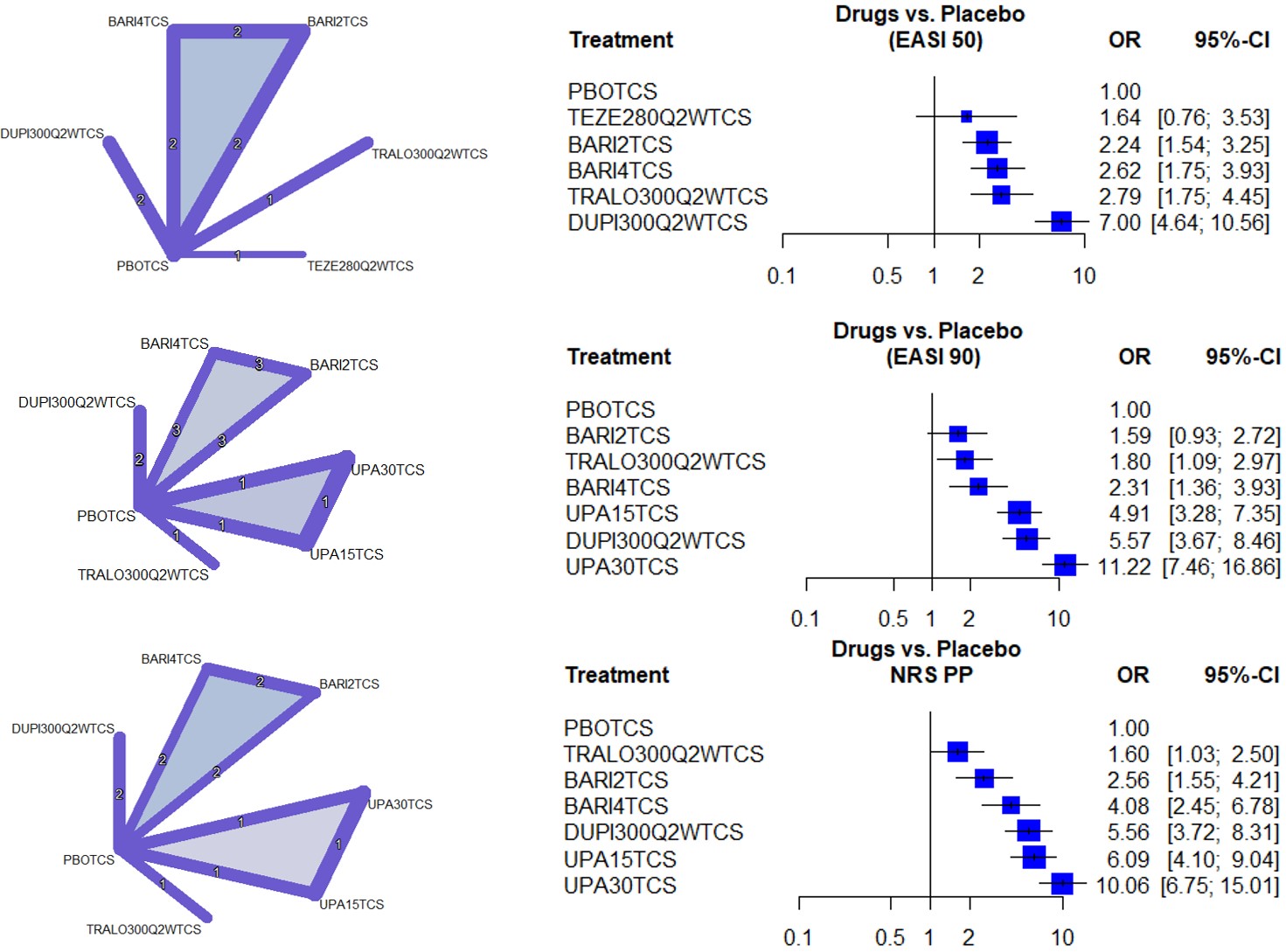

Supplement: Supplementary file 1 [file life-11-00927-s001.zip › FigureS4.jpg]

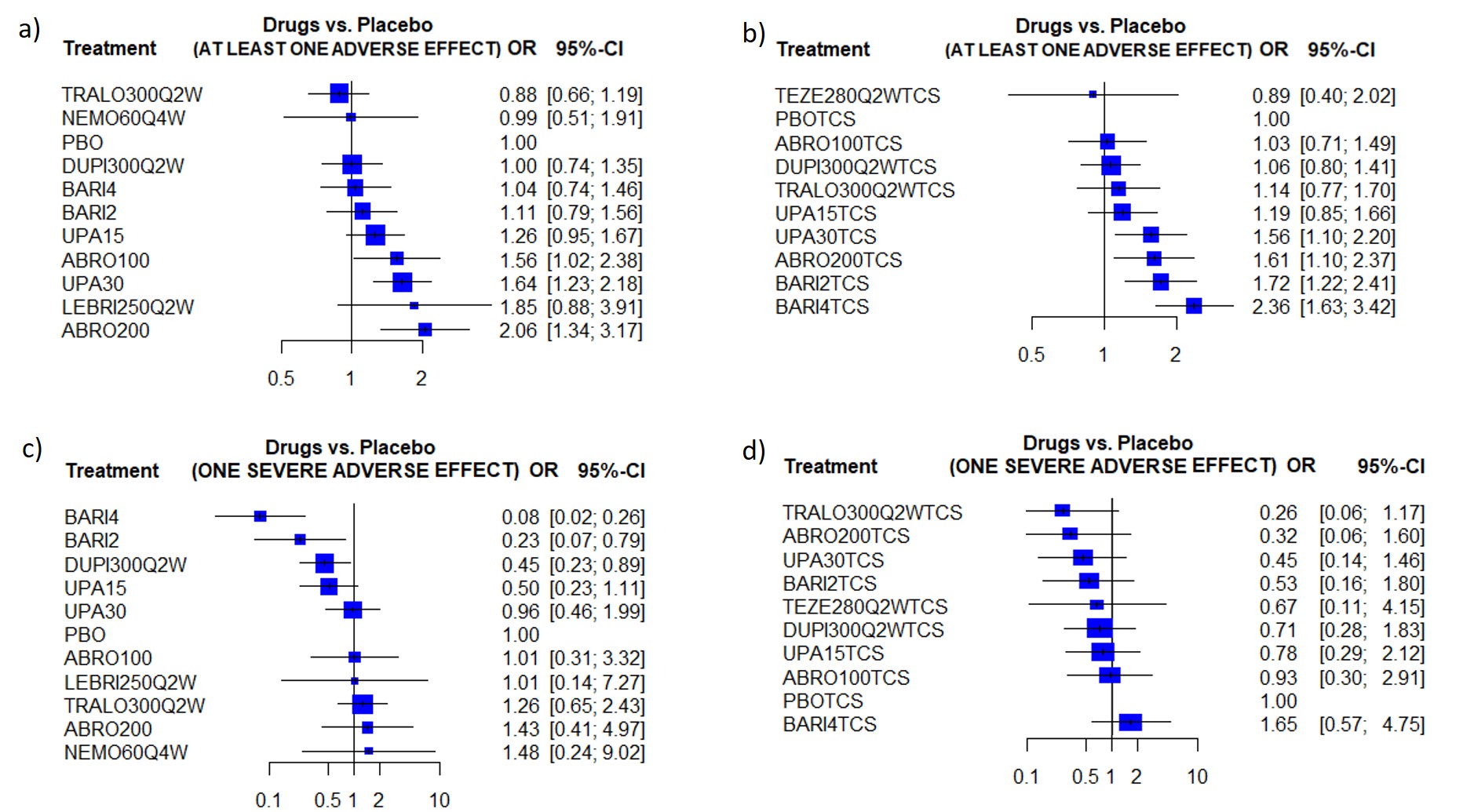

Supplement: Supplementary file 1 [file life-11-00927-s001.zip › FigureS5.jpg]
